# Metal-organic framework materials promote neural differentiation of dental pulp stem cells in spinal cord injury

**DOI:** 10.1186/s12951-023-02001-2

**Published:** 2023-09-04

**Authors:** Heng Zhou, Shuili Jing, Wei Xiong, Yangzhi Zhu, Xingxiang Duan, Ruohan Li, Youjian Peng, Tushar Kumeria, Yan He, Qingsong Ye

**Affiliations:** 1 Center of Regenerative Medicine, Department of Stomatology, Renmin Hospital of Wuhan University, Wuhan, 430060 China; 2 Terasaki Institute for Biomedical Innovation, Los Angeles, CA 90095 USA; 3 Institute of Regenerative and Translational Medicine, Tianyou Hospital of Wuhan University of Science and Technology, Wuhan, 430064 Hubei China; 4 Department of Oral and Maxillofacial Surgery, Massachusetts General Hospital, Harvard Medical School, Boston, MA 02114 USA; 5 School of Materials Science and Engineering, University of New South Wales, Sydney, NSW Australia

**Keywords:** Neural differentiation, Dental pulp stem cells, ZIF-8, MAPK, Spinal cord injury

## Abstract

Spinal cord injury (SCI) is accompanied by loss of Zn^2+^, which is an important cause of glutamate excitotoxicity and death of local neurons as well as transplanted stem cells. Dental pulp stem cells (DPSCs) have the potential for neural differentiation and play an immunomodulatory role in the microenvironment, making them an ideal cell source for the repair of central nerve injury, including SCI. The zeolitic imidazolate framework 8 (ZIF-8) is usually used as a drug and gene delivery carrier, which can release Zn^2+^ sustainedly in acidic environment. However, the roles of ZIF-8 on neural differentiation of DPSCs and the effect of combined treatment on SCI have not been explored. ZIF-8-introduced DPSCs were loaded into gelatin methacryloyl (GelMA) hydrogel and in situ injected into the injured site of SCI rats. Under the effect of ZIF-8, axon number and axon length of DPSCs-differentiated neuro-like cells were significantly increased. In addition, ZIF-8 protected transplanted DPSCs from apoptosis in the damaged microenvironment. ZIF-8 promotes neural differentiation and angiogenesis of DPSCs by activating the Mitogen-activated protein kinase (MAPK) signaling pathway, which is a promising transport nanomaterial for nerve repair.

**Supplementary Information:**

The online version contains supplementary material available at 10.1186/s12951-023-02001-2.

## Introduction

Globally, 250,000 to 500,000 people suffer from spinal cord injury (SCI) each year, mainly caused by traumatic events, as well as infections, tumors, and degenerative diseases [1]. Since damaged axons cannot be regenerated naturally in the adult central nervous system (CNS), SCI often leads to permanent sensory or motor dysfunction [1]. Primary injury causes compression or transection of the spinal cord, leading damage to nerve cells and disruption of the vasculature and blood-spinal cord barrier [2]. Subsequently, these mechanical damages induce a more terrible secondary injury cascade around the site. Edema and ischemia lead to further spinal cord swelling and compression, at the same time, inflammation and oxidative stress trigger additional apoptosis and poor post-injury microenvironment [3]. Surgery and various drugs such as glucocorticoids and gangliosides have shown limited effects in SCI clinical trials [4]. Therefore, there is an urgent need to develop novel and effective therapeutic strategies to improve neuronal axon regeneration and regulate the oxidative microenvironment to facilitate functional recovery after SCI.

In recent years, stem cell therapies have shown promising possibilities for the treatment of SCI, including mesenchymal stem cells (MSCs), neural stem cells (NSCs), and Schwann cells (SWs). Dental pulp stem cells (DPSCs), a kind of MSCs, has high proliferation potential and low immunogenicity, which are beneficial for allotransplantation. DPSCs originate from the neural ridge ectoderm and expresses more neural crista-related neural markers and neurotrophic factors, such as glial fibrillary acid protein (GFAP), beta III tubulin (βIII), brain-derived (BDNF) and glial neurotrophic factors (GDNF), than other MSCs [5]. This provides DPSCs with better neuroprotection and neurotrophic function to cope with nervous system injury. Some previous studies have reported that DPSCs as seed cells exhibit excellent therapeutic effects in rat SCI models. For example, an overexpression basic fibroblast growth factor of DPSCs engineered using hypoxic response elements has shown excellent angiogenesis for SCI repair [6]. A combination of N1-(4-boronobenzyl)-N3-(4-boronophenyl)-N1, N1, N3, N3-tetramethylpropane-1,3-diaminium and Laponite hydrogels with excellent reactive oxygen species (ROS) clearance and iron death inhibition, combined with DPSCs to regulate the ratio of excitatory and inhibitory synapses. The results show that the composite biomaterial can effectively reduce muscle spasms and promote the recovery of SCI [7].

Zinc is essential for the growth and development of human organs and tissues [8]. More than 300 enzymes require zinc to function properly, and zinc is therefore involved in regulating various cellular processes, including cell division and DNA synthesis [9]. Zinc is thought to interact with 10% of the human proteome, and it is essential for processes such as cell division and protein synthesis [10, 11]. More than 99% of intracellular zinc binds to proteins, and although there is growing evidence that interchangeable zinc ions act as second messengers capable of transducing extracellular stimuli into intracellular signaling events, the specific mechanisms remain uncertain [12]. The human body contains about 2–3 g of zinc, which is widely distributed in various tissues and organs of the human body. About 60% of them are in skeletal muscle, 30% are in bone, 5% are in liver and skin, and the rest are distributed in other tissues [13]. Zn^2+^ disorders are therefore associated with a variety of diseases, including high blood pressure [14], sudden cardiac death [15], cirrhosis of the liver [16], diabetes [17], osteoporosis [18], as well as anti-viral [19] and anti-bacterial deficiencies [20]. Zinc is closely related to neurogenesis. Most zinc chelate is contained in the presynaptic terminal vesicles of dentate granulosa cells forming mosses fiber bundles in the hippocampus, where neurogenesis and neural migration are active in the adult brain [21]. Extensive investigations have shown that zinc deficiency during pregnancy can impair neuronal differentiation and proliferation, leading to neurogenesis and cognitive dysfunction [22–24]. Although the exact mechanism is unclear, several studies have shown that zinc supplementation promotes in vitro neural differentiation of stem cells, including adipose-derived MSCs (AD-MSCs) [25] and induced pluripotent stem cells [26]. Zinc is known as an acute phase reactant due to its rapid redistribution from serum to the cell interval, and serum zinc concentrations are dramatically reduced in SCI animal models and clinical cases [27, 28]. Zinc has been associated with neuronal damage associated with traumatic brain injury (TBI), stroke, and epilepsy [29–31]. In addition, the loss of serum zinc concentration was negatively correlated with the long-term prognosis of motor function in SCI [30].

As a subfamily of metal-organic frameworks (MOFs), zeolitic imidazolate frameworks (ZIFs) present a zeolite-like crystal topology, which is characterized by large cavities connected by small windows [32]. Zeolitic imidazolate framework–8 (ZIF-8) with a topology of sodalite (SOD) is one of the most promising members of MOFs, given its great biocompatibility, high loading capacity and ROS scavenging activity [33]. Nanoscale ZIF-8 is very suitable for constructing pH-responsive functional scaffolds because of its stability under physiological conditions and decomposes when encountering acidic environments [34]. In addition to the drugs delivered by nano-carries, the Zn^2+^ released by ZIF-8 plays a vital role in regulating the differentiation of stem cells. ZIF-8 has been shown to promote osteoblast differentiation of bone marrow MSCs (BMSCs) through the classical mitogen-activated protein kinase (MAPK) pathway, providing a molecular basis for its application in bone tissue engineering [35, 36].

In this study, the roles of ZIF-8 in promoting neural differentiation of DPSCs have been revealed for the first time. ZIF-8 promotes neural differentiation and angiogenesis of DPSCs by activating the JNK1/p38 MAPK signaling pathway through the continuous release of Zn^2+^. In addition, ZIF-8 can help DPSCs resist apoptosis induced by Zn^2+^ deprivation and improve the survival rate of DPSCs. The therapeutic approach of ZIF-8 carried by DPSCs showed a favorable therapeutic effect in the rat SCI model.

## Results

### Synthesis and cellular uptake of ZIF-8 nanoparticles

**Fig. 1:**
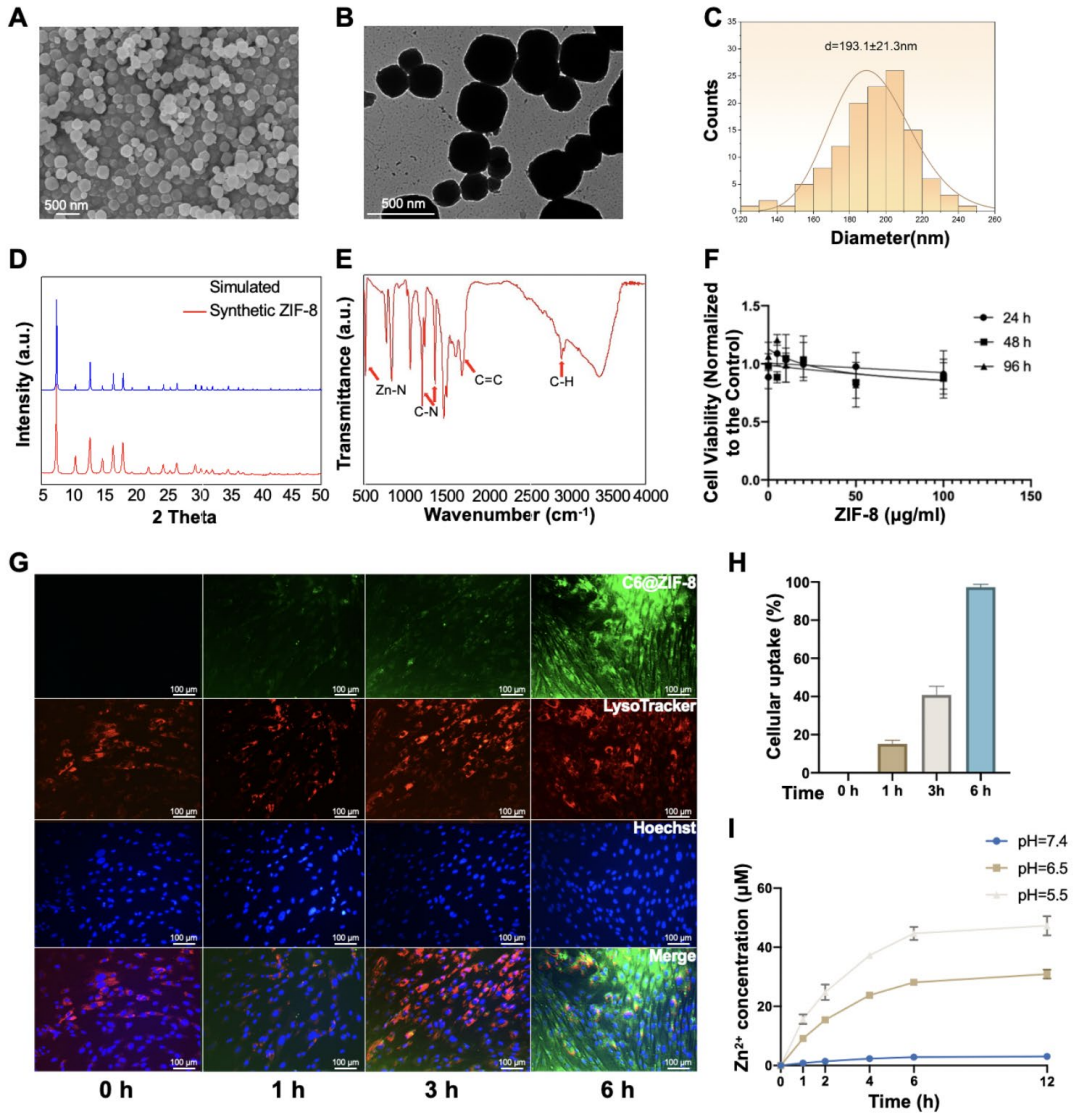
Characterization of nano ZIF-8. **(A)** SEM, **(B)** TEM, **(C)** Statistics of size, **(D)** PXRD, **(E)** FT-IR, **(F)** toxicological evaluation. Different concentrations (0, 5, 10, 20, 50 and 100 µg/ml) of ZIF-8 were added to DPSC for CCK-8 detection at 24,48 and 96 h **(G)** Intracellular trafficking of C6-labeled ZIF-8 (20 µg/ml; green fluorescence) in DPSCs. LysoTracker (red) was used to label the lysosome, and Hoechst 33,342 (blue) was used to label the nucleus. **(H)** Quantification of the relative fluorescence intensity of C6-labeled ZIF-8. ***P < 0.001. **(I)** ZIF-8 nanoparticles were dispersed in α-MEM with different PH values, and Zn^2+^ levels were detected at different time points

ZIF-8 nanoparticles were successfully synthesized using a previously reported one-pot method. Typical octahedral morphology of ZIF-8 was revealed by SEM and TEM in Fig. 1A and B, respectively. The prepared ZIF-8 nanoparticles have uniform size around 193.1 ± 21.3 nm (Fig. 1C). The powder X-ray diffraction (PXRD) analysis showed that the diffraction peaks of synthesized ZIF-8 were identical to the simulated one, which verified a high-purity phase of ZIF-8 (Fig. 1D). The Fourier-transform infrared (FT-IR) spectra indicated that the synthesized ZIF-8 was highly consistent with the previous crystals in peak position and peak shape, which further confirmed the successful synthesis of ZIF-8 (Fig. 1E) [35]. As expected, these nanoparticles had excellent cytocompatibility as measured by CCK-8 in DPSCs (Fig. 1F). The ZIF-8 nanoparticles were non-toxic to DPSCs up to 100 ug/mL concentration for up to 96 h. Immunofluorescent co-localization analysis showed that the green fluorescent ZIF-8 was largely colocalized with the red fluorescent lysosomes, indicating that ZIF-8 entered cells through endocytosis and accumulated in lysosomes (Fig. 1G). Within 6 h, the cellular uptake efficiency of ZIF-8 in DPSCs could reach up to 98.1% (Fig. 1H). The pH-sensitivity of ZIF-8 was confirmed by Zn^2+^ release assay. At physiological pH, the release of Zn^2+^ from ZIF-8 is very slow, however, when pH = 6.5 or pH = 5.5, it is greatly increased and lasts for several hours (Fig. 1I).

### ZIF-8 promotes neural differentiation of DPSCs

**Fig. 2:**
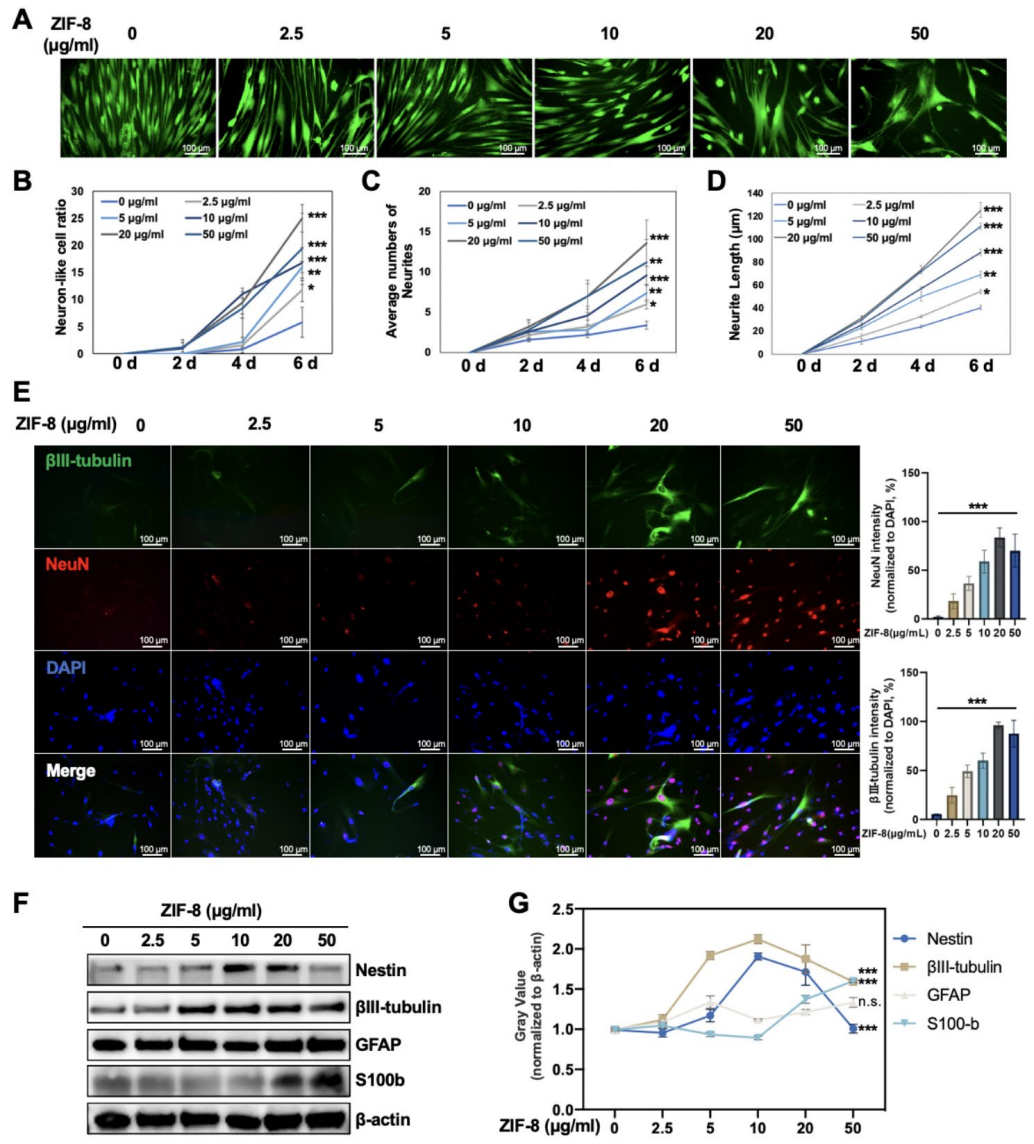
ZIF-8 promotes DPSC neural differentiation. **(A)** Fluorescent images of calcein-labeled DPSCs cells after different treatments of ZIF-8 (0, 2.5, 5, 10, 20 and 50 µg/ml) within B27 medium for 6 days. Trends of DPSCs neural differentiation (mean ± SD): **(B)** neural-like cell ratio, **(C)** the neurite number and **(D)** neurite length. DPSCs were treated with different concentrations of ZIF-8 (0, 2.5, 5, 10, 20 and 50 µg/ml) in B27 nerve differentiation culture for 7 days **(E)** Immunofluorescence analysis of expression of βIII-tubulin (green) and NeuN (red). Fluorescence intensity quantification of NeuN and βIII-tubulin is given. **(F)** Western Blot was used to detect the expression of Nestin, βIII-tubulin, GFAP, S100b and β-actin. **(G)** Quantitative statistics of gray value were given. ***P < 0.001

DPSCs are ideal seed cells for SCI treatment because they are more easily differentiated in nerve direction and express more neurotrophic factors. Flow cytometry showed that the surface antigens of extracted DPSCs were 87.4% CD73 + cells (Figure S1A), 99.2% CD44 + cells (Figure S1B), and 3.37% CD45 + cells (Figure S1C). DPSCs can be induced to differentiate into osteoblasts (Figure S1D) and lipoblasts (Figure S1E), respectively. Our previous research suggested that an important strategy for repairing nerve damage is to promote the neural differentiation of stem cells [3]. ZIF-8 can release Zn^2+^ in cells, which was reported to promote neural differentiation of IPSCs and AD-MSCs [25, 26]. To investigate the ability of ZIF-8 nanoparticles to induce neural differentiation, DSPCs were incubated with a concentration gradient of ZIF-8 (0, 2.5, 5, 10, 20, 50 µg/mL) within the B27 medium. The differentiating rate was examined and quantified every two days, as well as the length and the number of branching neurites. At the endpoint of the differentiation culture, the induction group with 20 µg/mL ZIF-8 showed the most desirable neurodifferentiation effect compared to the control group, followed by the group with 50 µg/mL ZIF-8 (Fig. 2A). The neurodifferentiation rate of DPSCs increased with increasing concentration of ZIF-8 in the range of 0 to 20 µg/mL and then decreased at 50 µg/mL (Fig. 2B). The number and length of neurites of DPSCs-derived neuron-like cells increased in a dose-dependent manner in the range of 0 to 20 µg/mL and they were significantly higher than those of the control group when the concentration of ZIF-8 was 20 µg/mL (Fig. 2C, D). Overall, ZIF-8 induced the differentiation of DPSCs into neuron-like cells. Next, we examined the expression of neural marker proteins by western blotting. The expression of βIII-tubulin and NeuN reflects the regeneration of early neuronal cells and mature neurons [37]. GFAP expression indicates glial scar formation [38]. S100b was used to evaluate the proliferation of Schwann cells, which is required for axonal regeneration after injury [39]. Immunofluorescence staining showed that the expression of βIII-tubulin and NeuN in the 20 µg/mL ZIF-8 group was significantly higher compared to the control group, suggesting that ZIF-8 at this concentration significantly promoted the differentiation of DPSCs into neuron-like cells (Fig. 2E). These results were confirmed via western blotting (Fig. 2F). ZIF-8 promoted the expression of neural markers such as βIII-tubulin, Nestin and S100b in DPSCs compared with the control group to varying degrees but did not change the level of GFAP (Fig. 2G). This indicates that ZIF-8 mainly promotes the differentiation of DPSCs into neurons rather than glial cells.

### ZIF-8 rescues apoptosis of Zn^2+^ deprivation

**Fig. 3:**
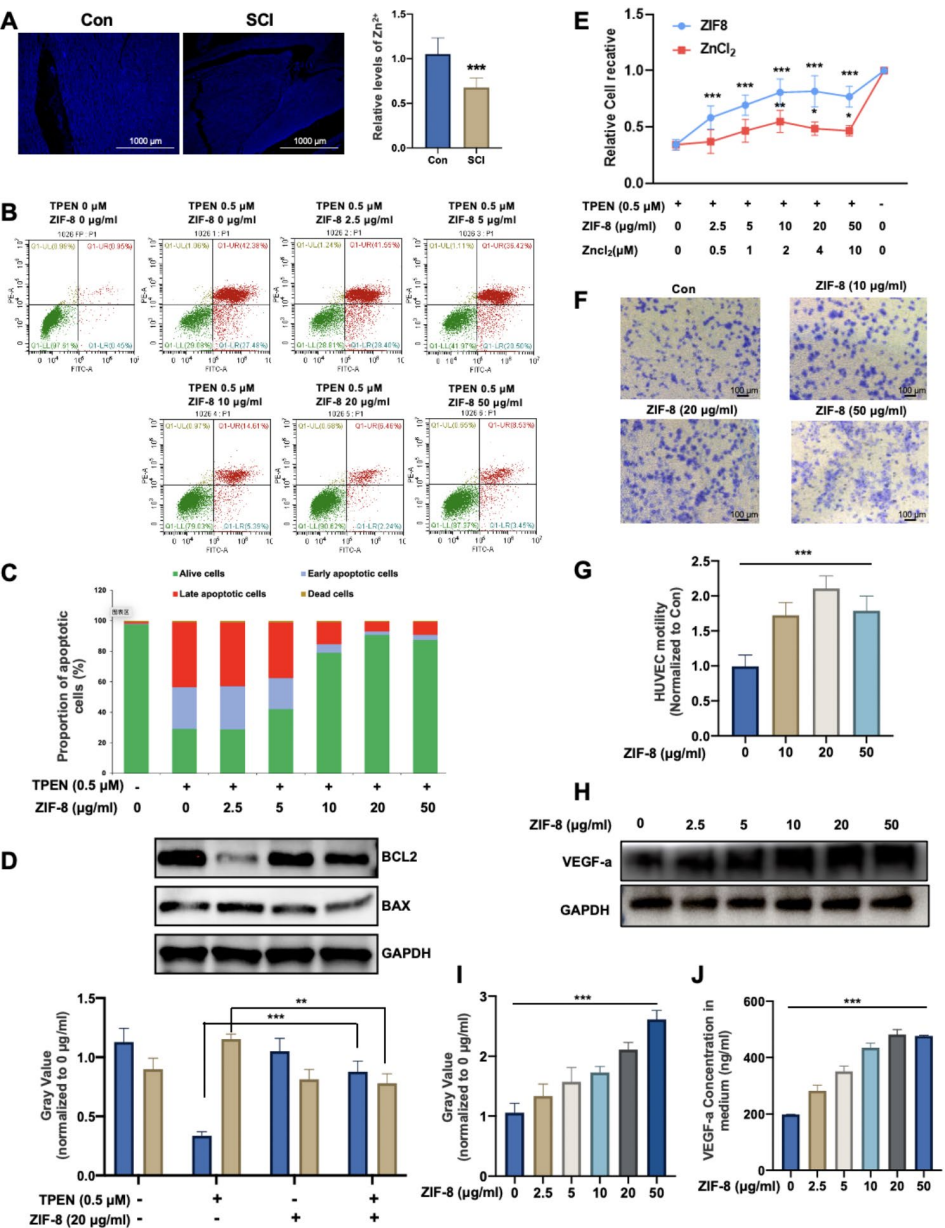
ZIF-8 contributes to resistance to apoptosis induced by Zn-deprivation and promotes angiogenesis of DPSCs. **(A)** TSQ fluorescence staining was used to detect Zn^2+^ in normal control (labelled as ‘Con’) or SCI rat spinal cord tissue cells (n = 6, mean ± SD); **(B)** DPSCs were treated with TPEN (0 and 0.5 µM) combined with ZIF-8 of concentration gradient (0, 2.5, 5, 10, 20 and 50 µg/ml). After 24 h treatment, flow cytometry was used to detect FITC and PE; **(C)** Statistical diagram of apoptotic cell distribution; **(D)** DPSCs were treated with TPEN (0 and 0.5 µM) combined with ZIF-8 of concentration gradient (0 and 20 µg/ml). Western Blot was used to detect Bcl2, BAX and GAPDH. Quantitative statistics of gray value were given. **(E)** DPSCs were treated with TPEN (0 and 0.5 µM) combined with ZIF-8 of concentration gradient (0, 2.5, 5, 10, 20 and 50 µg/ml) or Zncl_2_ (0, 0.5, 1, 2, 4 and 10 µM). After 24 h treatment, CCK-8 was used to detect cell viabilities. **(F)** After 72 h treated with ZIF-8 (0, 10, 20 and 50 µg/ml), the medium was co-culture with HUVECs. Then cells stained with the crystal violet stain for Transwell experiments. **(G)** The number of traversed cells is counted by the ImageJ software. **(H)** Western Blot of VEGF-a and GAPDH of DPSCs treated with ZIF-8 (0, 2.5, 5, 10, 20 and 50 µg/ml). Quantitative statistics of gray value **(I)** were given. **(J)** VEGF-a in the medium was quantified by ELISA. *P < 0.05; **P < 0.01; ***P < 0.001

SCI is accompanied by a severe reduction of Zn^2+^ levels in plasma [30]. To determine the bioactive Zn^2+^ levels at SCI sites, a Zn^2+^ special fluorescent probe — TSQ was applied. As shown in Fig. 3A, fluorescence quantification showed that the level of Zn^2+^ in the SCI model group was significantly lower than that in the Sham group, indicating that SCI caused a serious blow to the Zn^2+^ level in the post-injury microenvironment.

Zinc plays vital roles in various biochemical pathways and enzymes and participates in antioxidant defense and immune response, making it particularly important in the oxidative stress and inflammatory processes after SCI [40]. To determine the association between Zn^2+^ deprivation and survival of transplanted stem cells, we constructed an in vitro Zn^2+^ removal model by using N,N,N0,N0-tetrakis(2-pyridylmethyl)-ethylenediamine (TPEN), a Zn^2+^ chelator. As shown in Fig. 3B, TPEN significantly induced DPSCs apoptosis. However, the effect of TPEN-induced apoptosis was rescued by ZIF-8 treatment in a dose-dependent manner, and plateaued after 20 µg/mL (Fig. 3C). In addition, TSQ fluorescence showed that 20 µg/mL ZIF-8 could restore the intracellular Zn^2+^ to normal level (Figure S2A, S2B). Western blot also verified the reversal effect of ZIF-8 on TPEN-induced apoptosis in DPSCs. TPEN significantly inhibited the expression of antiapoptotic protein Bcl-2 and promoted the expression of the apoptotic protein Bax in DPSCs. After treatment of ZIF-8, the BAX and Bcl-2 expression level was restored (Fig. 3D). In addition, different concentrations of ZnCl_2_ and ZIF-8 were added to DPSCs with or without the treatment of 0.5 µM TPEN to detect the effect of Zn^2+^ on their viability. As shown in Fig. 3E, both ZnCl_2_ and ZIF-8 showed a significant effect against TPEN treatment. Importantly, ZIF-8 presented more effective than ZnCl_2_ due to its controlled release of Zn^2+^ (Fig. 3E). Taken together, these results suggested that ZIF-8 could contribute to DPSCs resist apoptosis induced by Zn^2+^-deprivation.

### ZIF-8 enhances the angiogenic effect of DPSCs

To determine whether ZIF-8 promotes the pro-angiogenesis of DPSC, human umbilical vein endothelial cells (HUVECs) were used in transwell migration assay (Fig. 3F). The results showed that more cells migrated through the membrane in the ZIF-8 groups, especially at the concentration of 20 µg/mL, indicating a strong angiogenic capacity of ZIF-8 (Fig. 3G). Western blotting showed significant upregulated expression of VEGF-a in DPSCs with increased concentrations of ZIF-8 (Fig. 3H and I). The concentration of VEGF-a in the supernatant of DPSCs treated with ZIF-8 for 3 days was quantified by ELISA, and the results indicated that ZIF-8 dose-dependently elevated the level of VEGF-a in the conditioned medium of DPSCs (Fig. 3J). In addition, cell migration from the transplant site to the injured ischemic area is an important step in stem cell therapy [41]. Transwell migration assay showed that more cells migrated across the membrane in the ZIF-8 group than in the control group. (Supplemental Fig. 2C and 2D). These results suggest that ZIF-8 enhances the expression and secretion of VEGF-a, thereby promoting the angiogenesis activity of DPSCs, as well as the migration ability.

### ZIF-8 enhances neural differentiation and pro-angiogenic effect of DPSCs through activating MAPK signaling pathway

**Fig. 4:**
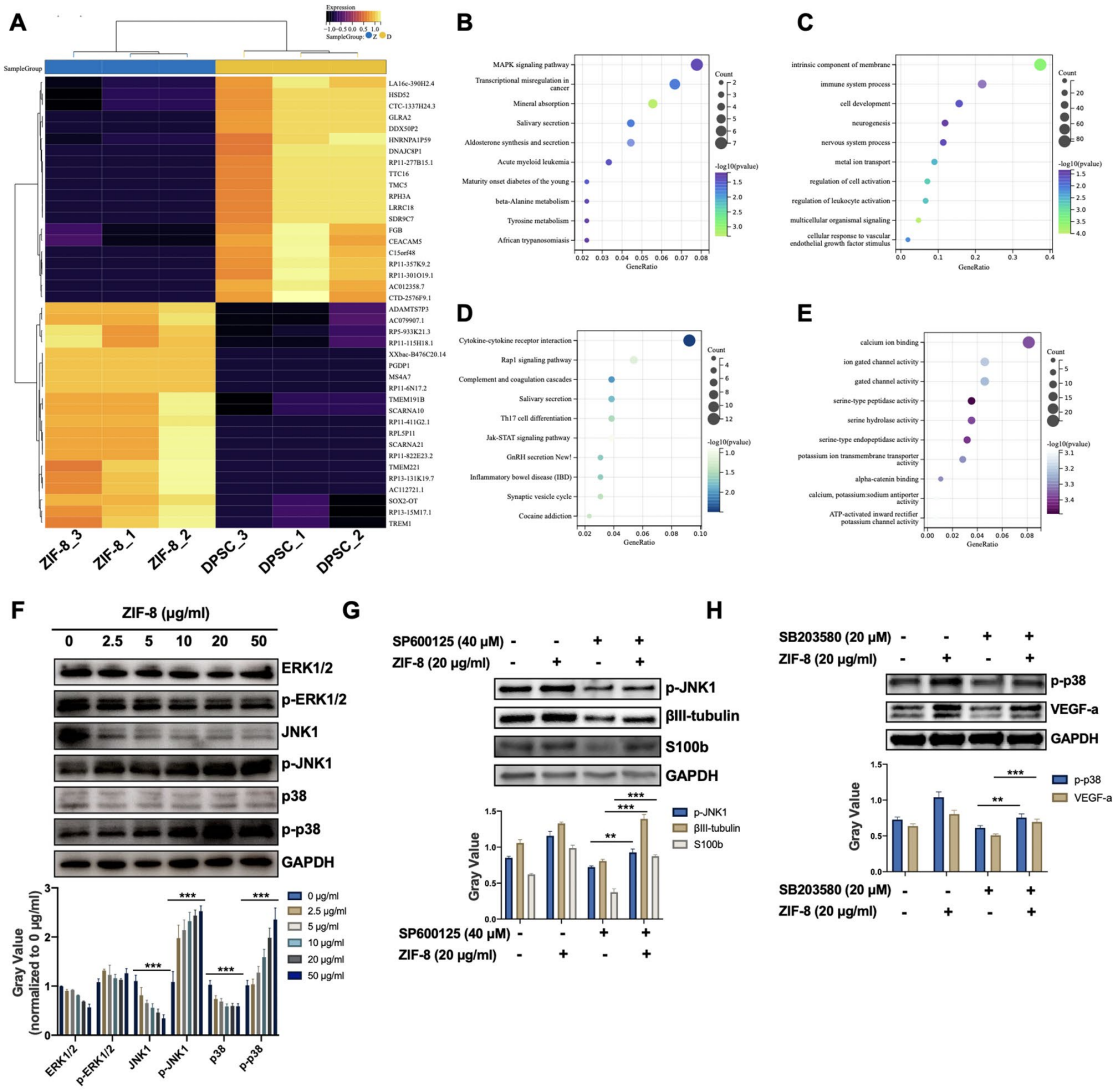
ZIF-8 promotes neural differentiation and angiogenesis of DPSCs through MAPK signaling pathways. **(A)** Heat map showing the top 40 differential gene expression. KEGG analysis of upregulated **(B)** and downregulated **(C)** signaling pathways. GO enrichment analysis of upregulated **(D)** and downregulated **(E)** signaling pathways. **(F)** DPSCs were treated with ZIF-8 (0, 2.5, 5, 10, 20 and 50 µg/ml) for 7 days in B27 medium. Western Blot was used to detect the expression of ERK1/2, p-ERK1/2, JNK1, p-JNK1, p38, p-p38 and GAPDH. Quantitative statistics of gray value were given. **(G)** DPSCs were treated with SP600125 (0 and 40 µM) combined with ZIF-8 (0 and 20 µg/ml). Western Blot was used to detect the expression of p-JNK1, βIII-tubulin, S100b and GAPDH. Quantitative statistics of gray value were given. **(H)** DPSCs were treated with SB203580 (0 and 20 µM) combined with ZIF-8 (0 and 20 µg/ml). Western Blot was used to detect the expression of p-38, VEGF-a and GAPDH. Quantitative statistics of gray value were given. **P < 0.01; ***P < 0.001

To further explore the mechanism by which ZIF-8 promotes neural differentiation and angiogenesis of DPSCs, second-generation sequencing was used to detect the changes of transcriptomic profiles. After 4 days of neurodifferentiation, a total of 2153 DEGs were identified, including 870 upregulated genes and 1283 down-regulated genes. The heat map shows the top 40 DEGs (Log2FC > 1.5, P < 0.05). These DEGs subsequently underwent KEGG and GO enrichment analysis (Fig. 4A). As shown in Fig. 4B, ZIF-8 promotes activation of MAPK signaling pathway, mineral absorption, and tyrosine metabolism, which are associated with neural differentiation. Go analysis indicated that ZIF-8 treatment was associated with the biological function of neurogenesis, nervous system process, cell development and immune system process, (Fig. 4C). The down-regulated genes were associated with Cytokine-cytokine receptor interaction, Rap1 signaling pathway, complement and coagulation cascade, etc. signaling pathways, as well as calcium ion binding, serine hydrolase activity and gated channel activity, etc. functional alternation (Fig. 4D F).

In view of the above analysis, the activation of the MAPK signaling pathway may play an important role in the biological activity of ZIF-8. Interestingly, under neural differentiation conditions, ZIF-8 inhibited the phosphorylation of ERK1 and activated the phosphorylation of two other MAPK key proteins —JNK and p38 (Fig. 4F). The phosphorylation of JNK1 and p38 is liable for the expression of βIII-tubulin and VEGF-a, respectively [42, 43]. This was demonstrated by an increased expression of p-JNK as well as βIII-tubulin and S100b (Fig. 4G) in presence of a specific p-JNK inhibitor, SP600125 when treated with ZIF-8. Similarly, another set of experiments showed an upregulated expression of p-p38 and VEGF-a induced by ZIF-8 could be reversed by SB203080, a p38 MAPK inhibitor (Fig. 4H). These results suggested that ZIF-8 promoted the neural differentiation and angiogenesis [43, 44] of DPSCs through JNK1/p38 MAPK pathways.

### ZIF-8 promotes neural differentiation and proangiogenic effects of DPSCs depends on continuous release of Zn^2+^

**Fig. 5:**
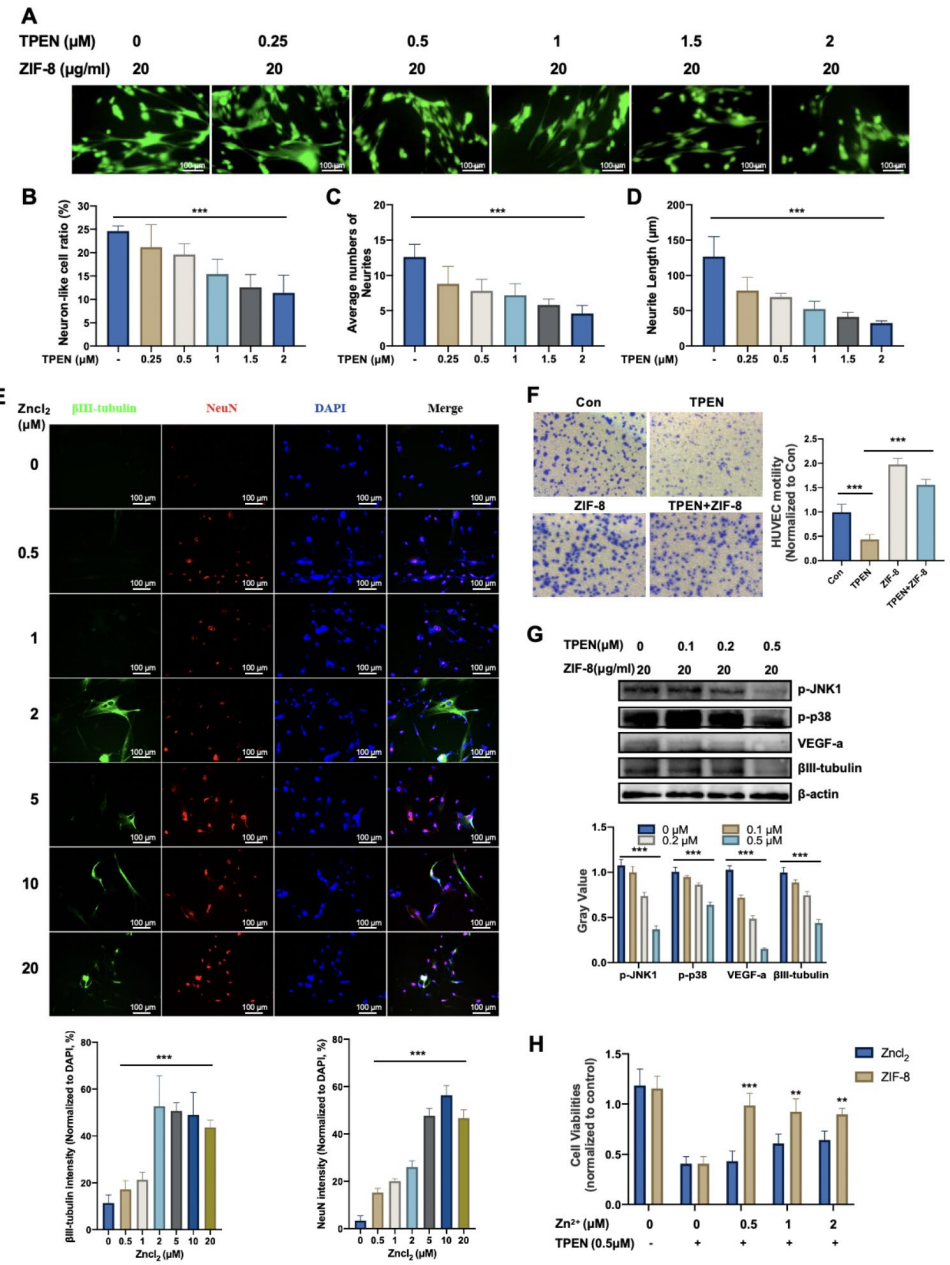
ZIF-8 promotes neural differentiation and angiogenesis of DPSCs depended on Zn^2+^ release. **(A)** Fluorescent images of calcein-labeled DPSCs cells treated with TPEN (0, 0.25, 0.5, 1, 1.5 and 2 µM) with ZIF-8 within (20 µg/ml) in B27 medium for 6 days. Trends of DPSCs neural differentiation (mean ± SD): **(B)** neural-like cell ratio, **(C)** the neurite number and **(D)** neurite length. DPSCs were treated with different concentrations of Zncl_2_ (0, 0.5, 1, 2, 4, 10 and 20 µM) in B27 nerve differentiation culture for 7 days **(E)** Immunofluorescence analysis of expression of βIII-tubulin (green) and NeuN (red). Fluorescence intensity quantification of NeuN and βIII-tubulin is given. **(F)** DPSCs were treated with TPEN (0 and 0.5 µM) combined with ZIF-8 of concentration gradient (0 and 20 µg/ml), the medium was co-culture with HUVECs. Then cells stained with the crystal violet stain for Transwell experiments. The number of transferred cells is quantified. **(G)** DPSCs cells treated with TPEN(0, 0.1, 0.25 and 0.5 µM) with ZIF-8 within (20 µg/ml) in B27 medium for 7 days. Western Blot was used to detect the expression of p-JNK1, p-38, VEGF-a, βIII-tubulin, and GAPDH. Quantitative statistics of gray value **(H)** were given. **(I)** DPSCs were treated with TPEN (0 and 0.5 µM) combined with ZIF-8 or Zncl_2_ of concentration gradient (0, 0.5, 1 and 2 µM). After 24 h treatment, CCK-8 was used to detect cell viabilities. **P < 0.01; ***P < 0.001

To explore whether ZIF-8 promotes neurogenesis of DPSCs by releasing Zn^2+^, varying amount of TPEN was added to the neural inducing medium containing 20 µg/ml ZIF-8. It was shown that ZIF-8-induced neural morphogenesis of DPSCs was disrupted by TPEN in a dose-dependent manner (Fig. 5A). The proportion of neuronal differentiation, the number and the length of neurites decreased in response to TPEN treatment (Fig. 5B, C, D). ZnCl_2_ was used to further certify whether Zn^2+^ can promote neural differentiation of DPSCs. As shown in Fig. 5E, ZnCl_2_ significantly upregulated the immunofluorescence intensity of βIII-tubulin and NeuN in DPSCs. To further compare the ability of ZIF-8 and ZnCl_2_ to promote neural differentiation, the same Zn^2+^ molar concentrations of ZIF-8 and ZnCl_2_ (1 and 2 µM) were treated with DPSCs. The results indicated that ZIF-8 was more potent than Zncl_2_ in inducing neural differentiation at the same molar concentration (Figure S3A and S3B). In addition, TPEN significantly downregulated the expression of p-JNK1, p-38, βIII-tubulin and VEGF-a with or without 20 µg/ml ZIF-8 treatment (Fig. 5G, Figure S3C). Regarding angiogenesis, ZIF-8 significantly increased the number of HUVEC migrating across the membrane, which was reduced with the action of TPEN (Fig. 5F). In addition, the ELISA assay showed that the extracellular secretion of VEGF-a stimulated by ZIF-8 can be reversed by TPEN (Figure S3D). Cell viability assay showed that ZnCl_2_ could rescue TPEN-induced apoptosis, which is inferior to ZIF-8 at the same molar concentration (Fig. 5H). These results suggested that ZIF-8 promotes neural differentiation and proangiogenic effect of DPSCs depends on releasing Zn^2+^.

### ZIF-8-DPSCs promote motor function recovery in rats after SCI

**Fig. 6:**
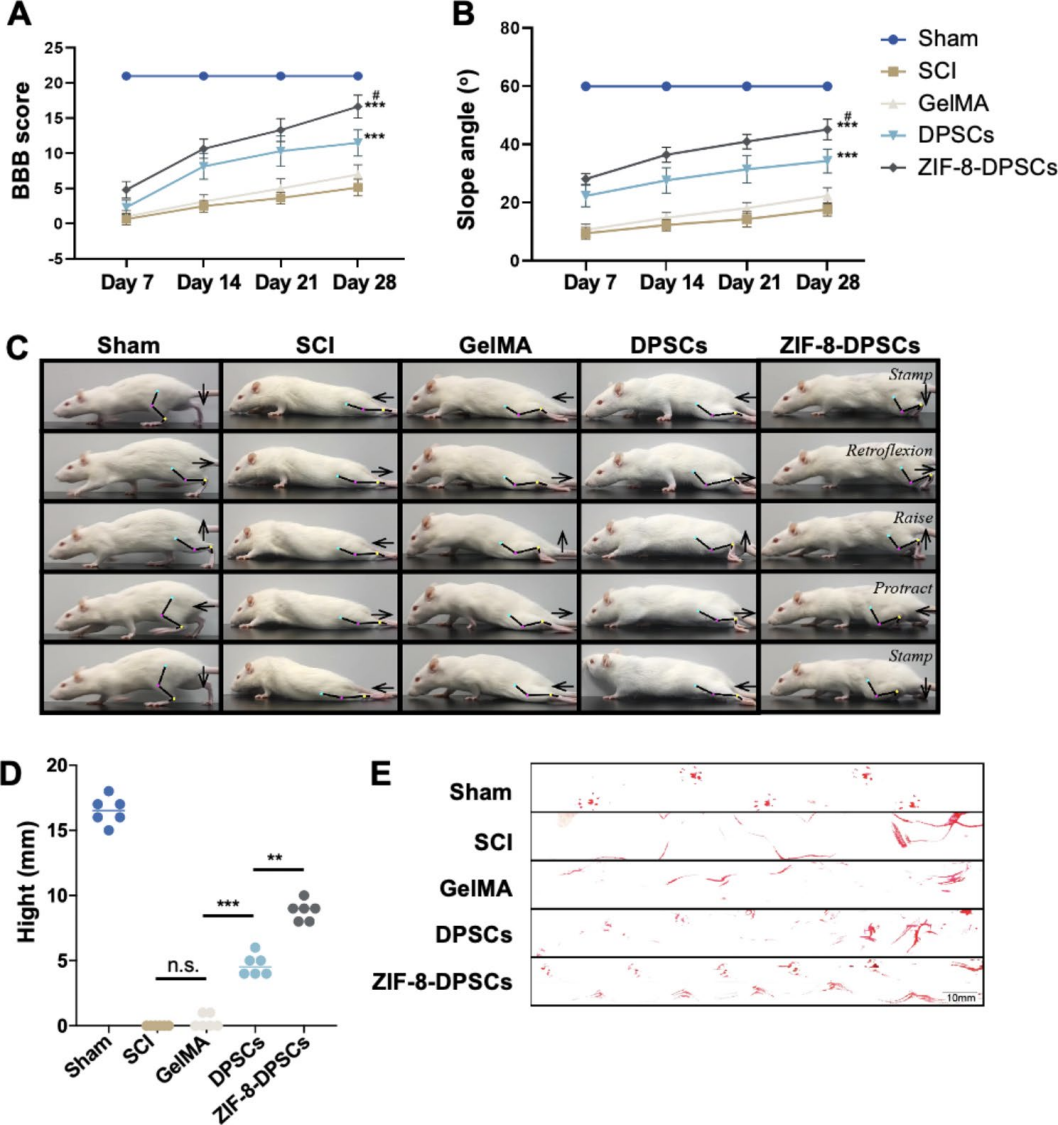
ZIF-8-DPSCs promote the recovery of motorial function in SCI rats. **(A)** The BBB scores of the rats in the Sham, SCI group, GelMA group, DPSCs group and ZIF-8-DPSCs group. **(B)** The inclined plane test scores of rats in the Sham, SCI group, GelMA group, DPSCs group and ZIF-8-DPSCs group. **(C)** Video analysis showed the rats walking at a speed of 28 d.a.t. Weight support, leg extensor spasm, walking, and foot placement were analyzed. Foot movements are highlighted by arrows. The initial position of the move is highlighted by the yellow dot. **(D)** Weight support (quantified as the height of the trunk from the ground). **(E)** Footprint analysis of Sham, SCI group, GelMA group, DPSCs group and ZIF-8-DPSCs group in 28 d.a.t. **P < 0.01. *** P < 0.001. n.s. means no statistical significance. # P < 0.05 compared with DPSCs group. Data are represented as mean ± SD (n = 6)

The motor functions of rat hind limbs were assessed on day 7, 14, 21, 28 using Basso-Beattie-Bresnahan (BBB) score, slope test, gait, and footprint analysis. There was no significant difference in BBB scores between the groups in one week after the operation. At subsequent time nodes, the BBB score of the ZIF-8 + DPSCs group was significantly improved, followed by the DPSCs only group (Fig. 6A). In the slope test, rats in the ZIF-8 + DPSCs group could stay at a greater angle than those in the SCI group and other treatment groups (Fig. 6B). At day 28 after the operation, the rats of the SCI model group had no obvious hindlimb activity, and the connection lines of joints were roughly horizontal and straight. In the GelMA group, the hindlimb motor function of the rats was not improved significantly, and only subtle minor joint movements were observed. The rats in the DPSCs group could move their hind limbs. The ZIF-8-DPSCs group showed complete hind limb lift and forward stride, although there was a certain gap with the Sham group (Fig. 6C). Compared with the SCI group and other treatment groups, rats in ZIF-8-DPSCs group had higher height from the ground (Fig. 6D), less foot error (Figure S4A), shorter spasm duration (Figure S4B) and longer forward distance (Figure S4C). The pads of the sham group showed clear footprints equidistantly arranged in two straight lines. However, those of the SCI group and GelMA group showed obvious passive dragging marks. Though the DPSCs group showed initial off-ground movement, the rats in the ZIF-8 + DPSCs group showed the best movement outcome with two orderly linear footprints. Although considerably improved, it is worth noting, that the rats in this group were still dragging tails and had a shorter stride distances (Fig. 6E).

**Fig. 7:**
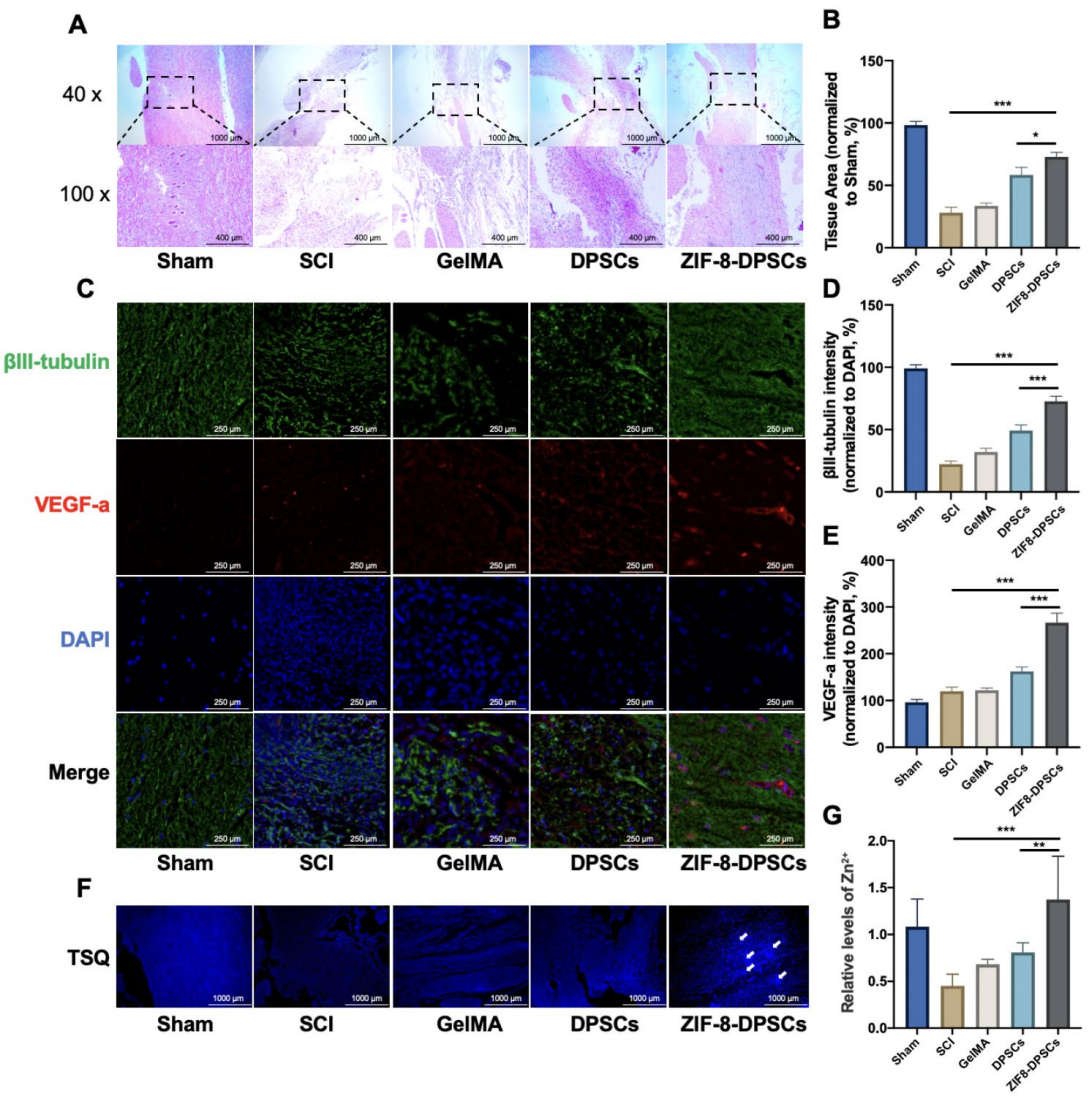
ZIF-8-DPSCs promote nerve tissue formation in SCI rats. **(A)** H&E staining transverse section of injured spinal cord tissue 28 days after injury. Above is the spinal cord at low magnification, with scar tissue and cavity visible in the SCI group, and below is the spinal cord at higher magnification. **(B)** Spinal cord tissue preserved on day 28 after spinal cord injury. Quantitative analysis of HE sections showed that. **(C)** The expression of VEGF-a and βIII-tubulin in the Sham, SCI group, GelMA group, DPSCs group and ZIF-8-DPSCs group was analyzed by immunofluorescence. Fluorescence intensity quantification of βIII-tubulin **(D)** and VEGF-a **(E)** is given. **(F)** TSQ fluorescence staining was used to detect Zn^2+^ in the Sham, SCI group, GelMA group, DPSCs group and ZIF-8-DPSCs group. The white arrow points to cells that overexpress Zn^2+^. **(G)** Fluorescence intensity of Zn^2+^ is quantified. *P < 0.05. **P < 0.01. *** P < 0.001. Data are represented as mean ± SD (n = 6)

Four weeks after the operation, the spinal cord segments around the injury site were dissected for histological analysis. H&E staining showed loose cells in the SCI group and much dense tissue in the ZIF-8-DPSCs group (Fig. 7A). Compared with the control SCI group, the normal tissue area of the ZIF-8-DPSCs group increased significantly (Fig. 7B). Double staining with βIII-tubulin and VEGF-a was used to analyze the regeneration of nerves and blood vessels at the injury site (Fig. 7C). The expression of βIII-tubulin was significantly increased in the ZIF-8-DPSCs group compared with the SCI group, indicating that ZIF-8-DPSCs have strong neural repair effects (Fig. 7D). The expression of VEGF-a was significantly increased in the ZIF-8 + DPSCs group compared with the Sham and SCI groups, indicating the angiogenic potency of ZIF-8 + DPSCs (Fig. 7E). IHC staining verified the activation of JNK and p38 MAPK pathways by ZIF-8 (Figure S5). Quantitative analysis showed that the expression of p-JNK1 and p-p38 in ZIF-8-DPSCs group was significantly improved compared with that in SCI group (Figure S5B, S5C). To verify whether Zn^2+^ is responsible, Zn^2+^ levels were assessed using TSQ. The fluorescence image of the SCI group was darker than that of the Sham group, indicating a decrease of the Zn^2+^ level during SCI (Fig. 7F). Quantitative analysis showed that Zn^2+^ in the ZIF-8 + DPSCs group was restored, even exceeding the Sham group (Fig. 7G). Interestingly, some concentrated Zn^2+^ concentration regions of cells could be found in the tissues from ZIF-8 + DPSCs group after 28 days of repairment (white arrows). Nissl staining images showed that the number of Nissl’s body in the injured spinal cord of rats in the ZIF-8-DPSC group was increased (Figure S6A and B). Tunel staining demonstrated that apoptosis in the ZIF-8-DPSCs group was inhibited compared with the SCI group (Figure S6A and C). In summary, ZIF-8-DPSCs significantly improved the recovery of motor function and repairment of neural tissues in rats after SCI.

### The cytotoxicity assessment of ZIF-8-DPSCs in vivo

Although the in vivo safety of ZIF-8 nanoparticles or DPSCs has been recognized, the safety of its combination needs to be examined. After 28 days of treatment, the rats were subjected to H&E and TSQ staining of major organs and hematological analysis to determine systemic toxicity of ZIF-8-DPSCs. H&E staining showed that the cell morphology and tissue structure of the major organs included lung, liver, spleen, kidney and heart were not changed by ZIF-8-DPSCs treatment (Figure S7A). Because ZIF-8-DPSCs treatment altered Zn^2+^ levels in local SCI tissues, Zn^2+^ levels in other major organs were detected. TSQ results suggested that ZIF-8-DPSCs did not significantly enhance the level of Zn^2+^ in other tissues. In addition, typical Zn^2+^ overexpression cells in local SCI tissues were not found in other organs (Figure S7B). Interestingly, treatment with ZIF-8-DPSCs and DPSCs both promoted weight gain in rats (Figure S7C). There was no significant difference in ALT and AST levels between the groups, indicating that ZIF-8-DPSCs did not have significant hepatotoxicity (Figure S7D and S7E). Levels of CREA and UREA were significantly increased in the SCI group. However, treatment with ZIF-8-DPSCs rescued the CREA and UREA levels suggesting the protective effect on the kidneys (Figure S7F and S7G). Similar to histological results, ZIF-8-DPSCs increased serum Zn^2+^ levels in SCI rats (Figure S7H). These results suggest that ZIF-8-DPSCs have good biocompatibility.

## Discussion

ZIF-8 is often used in the field of biomedicine as a drug or gene carrier. Interestingly, ZIF-8 has been less studied as a nanoparticle to present biological functions. In this paper, we propose for the first time that ZIF-8 can promote neural differentiation of DPSCs by activating the MAPK signaling pathway through the release of Zn ions. ZIF-8 promoted the expression of VEGF-a and the angiogenic ability of DPSCs. In addition, ZIF-8 can resist the apoptosis of DPSCs caused by Zn^2+^ deprivation. In vivo experiments, ZIF-8 combined with DPSCs and GelMA hydrogel promoted the recovery of motor function in SCI rats. Tissue slides showed that ZIF-8-DPSCs promoted neural recovery, antiapoptotic and angiogenic function of SCI rats.

SCI is closely related to serum zinc ion concentration. An important correlation is that acute SCI is accompanied by a sharp decrease in serum Zn^2+^ concentration. Heller et al. reported a 30% decrease in serum Zn^2+^ concentration in SCI patients at the acute injury stage [28]. This finding was further confirmed in the SCI mouse model [45, 46]. More importantly, acute serum zinc concentrations decreased proportionally with the severity of SCI [28]. Our study found that in the pathological injury of chronic convalescent rats with SCI, intracellular Zn ions were significantly deprived. Mechanistically, monocytes are activated rapidly in response to the intense inflammatory response to injury[30]. Through the TLR4/NF-κΒ/ZIP8 signal pathway, mononuclear cells absorb the serum Zn^2+^ concentration, and transfer it into the damaged tissues[47–49]. Secondary injury of SCI is often a more important cause of neuron loss. Glutamate excitatory toxicity is a common mechanism of neuronal tissue death at this stage [50]. Reduced extracellular Zn^2+^ concentration induced by trauma can lead to dysregulation of the NF-κB signaling pathway, which leads to glutamate excitotoxicity of astrocytes and promotes apoptosis of oligodendrocytes[51]. In zinc deficiency conditions, oligodendrocyte exposure to glutamate causes toxicity, but not at sufficiently high zinc concentrations, highlighting the importance of zinc in preventing glutamate excitotoxicity. High levels of zinc inhibit Ca^2+^ current through AMPA (α-amino-3-hydroxy-5-methyl-4-isooxazopropionic acid) receptors, suggesting that Zn^2+^ inhibits charge current through ionic glutamate receptors[52]. This result is consistent with our findings. In order to mimic the environment of Zn^2+^ loss in SCI, we constructed an in vitro model of Zn^2+^ deprivation. With the addition of TPEN, Zn^2+^ in DPSCs was significantly inhibited. Cell apoptosis was obviously induced and cell activity decreased. This indicated that Zn^2+^ deprivation significantly inhibited the survival of transplanted stem cells. With the increase of ZIF-8 concentration, the pro- portion of apoptosis decreased and the cell activity increased. Zn2 + supplementation also reversed the TPEN-induced inhibition of cell viability and apoptosis. In addition, ZIF-8 combined with DPSCs can better repair SCI damage in vivo, promote the occurrence of local neurons and blood vessels, and inhibit the apoptosis of damaged local cells.

Currently, a consensus of cell therapy is that the use of scaffolds to concentrate stem cells at the injured site and form a 3D repair environment is conducive to the differentiation and survival of stem cells. Porous gelatin methacryloyl (GelMA) hydrogels with properly connected micropores have a good cellular response and can promote neural stem cell migration, which is suitable for the repair of SCI alone [53] or in combination with seed cells[54, 55]. In this study, the motor function repair potential of ZIF-8 combined with DPSCs after SCI was assessed by transplanting ZIF-8 and DPSCs (labelled ZIF-8-DPSCs) loaded with GelMA hydrogel at the SCI site. We tried to use GelMA hydrogel as scaffold material and DPSCs as seed cells to repair SCI, and the results were similar to those of other studies. The basic logic is that we have successfully used this combination to repair large sciatic nerve defects in rats[56].

Our previous research suggests that an important strategy for repairing nerve damage is to promote the neural differentiation of stem cells. Wan and his colleagues proposed that ZIF-8 promotes osteogenic differentiation of BMSC and demonstrated this in mouse models of skull and mandible defects. They suggested that ZIF-8 could promote the activation of the classical MAPK signaling pathway, promoting the phosphorylation of extracellular signal-regulated kinase (ERK) 1/2 and the expression of RUNX2. However, the mechanism of action of ZIF-8 is not entirely dependent on the release of Zn^2+^[35, 57]. MAPK pathway is important for cell proliferation, differentiation, and apoptosis, and it includes ERK, C-Jun N-terminal kinse/stress-activated protein kinase (JNK/SAPK) and p38 kinase[58]. The role of the MAPK family in neural differentiation of stem cells has been investigated. JNK regulates the neural differentiation of PC12 cells and mice embryonic stem cell (mESC)[59]. Seong et al. found that Toll-like receptor 5 (TLR5) promoted the expression of βIII-tubulin in neural stem cells by promoting the phosphorylation of JNK1/2. Under differentiated conditions, the JNK pathway inhibitor SP600125 significantly reduced the number of βiii tubulin positive cells and decreased p-JNK expression in TLR5 KO or TLR5 shRNA transfected neuro-globules during neural differentiation compared to WT mice[42]. Here our results suggested a different conclusion: under the condition of nerve differentiation culture, ZIF-8 significantly promoted the number neuron-like cells from DPSCs, the number and length of axons, and promoted the expression of neural markers such as Nestin, βIII-tubulin, S100b, etc. In addition, we found that ZIF-8 activated different MAPK signaling pathways, including phosphorylation of JNK1/2 and p38. The role of the MAPK family in neural differentiation of stem cells has been investigated.

ZIF-8 will gradually release Zn^2+^ in cells under acidic environment, which is suitable for local weak acidic environment of SCI[7, 60]. Cellular zinc homeostasis plays an important role in iPSC differentiation, and zinc deficiency significantly impairs iPSC neuronal differentiation[61]. Zn^2+^ supplementation can upregulate the expression of Nestin and promote the neural differentiation of iPSCs[26], as well as AD-MSCs[25]. This role in promoting neural differentiation depends on classic ERK1/2 MAPK signaling pathway activation, which is different from our finding that ZIF-8 activates JNK1 phosphorylation in DPSCs. A few works have reported that the high concentration of Zn^2+^ can activate the JNK1 phosphorylation referring to different physiological processes[62, 63]. Combination with our results that Zn^2+^ deprivation inhibited expression of neural biomarkers both with or without ZIF-8 treatment, the mechanism by which ZIF-8 induces DPSCs neural differentiation maybe depending on the activation of JNK1 through the release of Zn^2+^ to stimulate the expression of βIII-tubulin and other downstream neural markers. Importantly, compared with direct Zn^2+^ supplementation, ZIF-8 at the same molar concentration has a better ability to promote neuronal differentiation and resist Zn-deprivation induced cell death. We speculated that this might be due to the continuous release of Zn^2+^ by ZIF-8 in cells. Interestingly, Zn^2+^ overexpressed cells were found to be survived in SCI tissues until the rat sacrifice.

## Conclusion

**Fig. 8:**
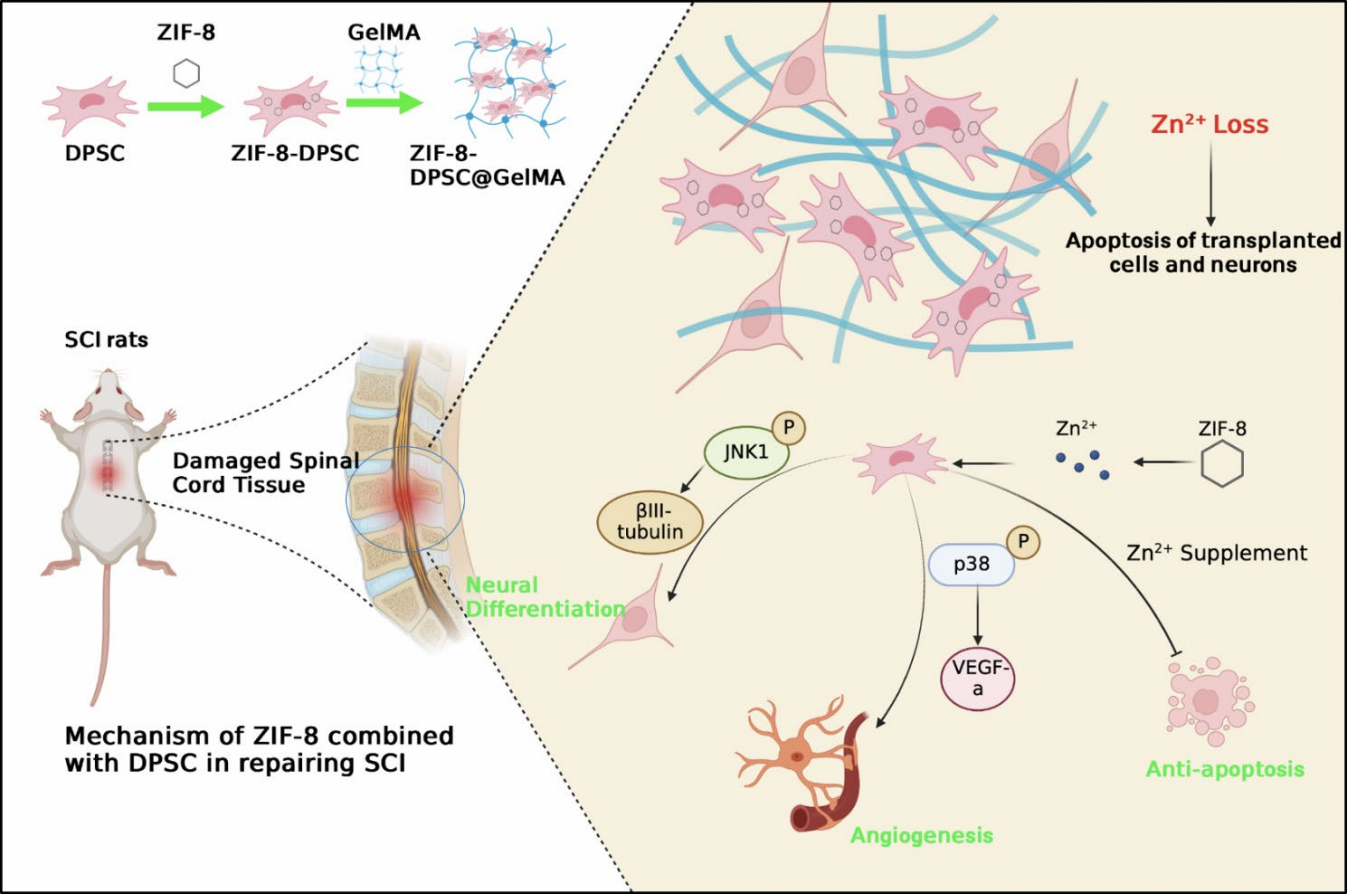
Schematic diagram of ZIF-8 combined with DPSCs to repair spinal cord injury. Loss of Zn^2+^ at the site of spinal cord injury leads to apoptosis of local transplanted stem cells and neurons. ZIF-8 slowly releases Zn^2+^ in DPSCs, helping to resist apoptosis induced by Zn deprivation. In addition, Zn^2+^ promotes the phosphorylation of JNK1 to upregulate the expression of βIII-tubulin, and promotes the neural differentiation of DPSCs. Phosphorylation of P38 also leads to increased expression of VEGF-a and promotes angiogenesis

In conclusion, the bioactive function of promoting stem cell neural differentiation by ZIF-8 nanoparticles was firstly proposed. ZIF-8 induces neurodifferentiation and promotes the angiogenesis of DPSCs by activating JNK1 and p38 MAPK pathways. Compared with direct Zn^2+^ supplementation, ZIF-8 at the same molar concentration has a better ability to promote neuronal differentiation and resist Zn-deprivation induced cell death. When ZIF-8-DPSCs@GelMA was implanted into the SCI site, ZIF-8 compensated for the Zn^2+^ loss caused by injury, and promoted the neural differentiation and VEGF-a secretion of DPSCs, promoting vascularity recovery and nerve regeneration (Fig. 8). Our findings provided the fundamental evidence for the future application of ZIF-8 as a carrier tool involved in nerve injury repair.

## Materials and methods

### Preparation and identification of nano ZIF-8

ZIF-8 nanomaterials were prepared by reference to the previous report[64]. Specifically, equal volumes of 25.6 mM 2-methylimidazole (2-MIM) methanol solution and 25.2 mM Zn(NO_3_)_2_ aqueous solution were mixed and kept for 15 min. After ultrasonication, white precipitates were collected by centrifugation (4 °C, 12,000 rpm, 10 min). These primary powders were washed thrice with PBS and then vacuum dried to obtain final ZIF-8 nanoparticles, which were stored at low temperature and dissolved in PBS for use. Transmission electron microscopy (TEM, HITACHI, HT7700, Japan) and scanning electron microscopy (SEM, Zeiss SIGMA, UK) were used to observe the microstructures of ZIF-8. Grain sizes were measured using ImageJ software (1.53a) and plotted using Origin software (8.0). XRD patterns of nano-ZIF-8 were acquired using the D4 ENDEAVOR X-ray diffractometer (PANalytical, XPert Pro, Netherlands). FT-IR spectra of nano-ZIF-8 in the range of 400–4000 cm^− 1^ were obtained on Thermo Fisher Scientific FT-IR spectrometer (Nicolet 5700, USA) using potassium bromide disks.

### Extraction and identification of DPSCs

DPSCs were cultivated and identified according to our previous developed protocol[56]. Briefly, impacted third molars from healthy individuals (15–25 years old) were collected from the Department of Stomatology, Renmin Hospital of Wuhan University. The study was approved by the Ethics Committee of Renmin Hospital of Wuhan University and informed consent was obtained from all donors. After cleaning and disinfecting the tooth surface with alcohol, the teeth were dissected aseptically, and the pulp tissue was isolated and rinsed in PBS. The dental pulp tissue was cut into small pieces of 1mm^3^, and digested with type I collagenase and dispase (Sigma, Germany) at 37 °C for 1 h with shaking once every 10 min. After termination of digestion, the cell pellet was resuspended with α-modified Eagle’s medium (α-MEM, Gibco, USA) containing 20% fetal bovine serum (FBS, Gibco, USA), 100 µg/mL streptomycin, and 100 U/mL penicillin (Gibco, USA) and incubated in T-25 culture flasks in a standard culture environment (37 °C, 5% CO_2_). The culture medium was changed every 3 days. Cells from passages 3 to 5 were used for experiments.

### Intracellular uptake of ZIF-8

With reference to the previous method[34], coumarin 6 (C6) was fully mixed with 2-MIM solution before adding Zn(NO_3_)_2_. DPSCs were seeded in a 12-well cell culture plate and cultured for 24 h. Then, these cells were labeled with LysoTracker (red) for lysosomes and Hoechst 33,342 (blue) for nucleus, and C6-labeled ZIF8 (20 µg/mL, green) was added into the medium. At 0, 1, 3, and 6 h, real-time living cell imaging were captured by inverted fluorescence microscope (Leica, DMi8, Germany). To measure the rate of Zn^2+^ release, ZIF-8 was dispersed in α-MEM with different PH values. The Zn^2+^ levels were detected at different time points by a kit method (Elabscience, China) using a microplate reader (PerkinElmer, EnSight, USA).

### Cell viability assay

The cytotoxicity of ZIF8 nanomaterials was tested by Cell Counting Kit-8 (CCK-8, MCE, USA) assays. DPSCs were seeded in 96-well cell culture plates at a density of 5000 cells/well and cultured overnight. ZIF-8 was added into the medium at a concentration gradient of 0,2.5,5,10,20,50 µg/mL, and these cells were further incubated for 24 h. After washing twice with PBS, 100 µL of freshly prepared CCK-8 detection solution was added to each well. After incubation at 37 °C for 2 h, the absorbance was measured at 450 nm by a microplate reader (PerkinElmer, EnSight, USA). The same method was used to determine the appropriate concentration of TPEN (MedChemExpress, HY-100,202) chelating zinc ions in DPSCs.

### Cell apoptosis analysis

The flow cytometric analysis was used to determine The recovery ability of ZIF-8 to TPEN-induced apoptosis. Briefly, the DPSCs were pretreated with a concentration gradient of ZIF-8 overnight, and then TPEN (2 µM) was added into the medium. After 24 h, the control and treated cells were collected and counted. According to the specification of the detection kit (Beyotime, C1062), 10^6^ cells were taken and stained with annexin V and PI. The apoptosis was analyzed applying a Beckman CytoFLEX S flow cytometer (Beckman Coulter, CA, USA).

### Measurement of neurite growth

Based on previous methods[65], DPSCs were treated with different concentrations of ZIF-8 (0–50 µg/mL) and differentiated in B-27 Plus Neuronal Culture System (Gibco, A3653401) for 6 days. The cells were stained with Calcein AM(Beyotime, C2012)according to the recommended experimental method. The cell morphology and neurite length were observed using an inverted fluorescence microscope.

To investigate the roles of zinc ions in the neural differentiation of DPSCs, the cells were treated with a concentration gradient of TPEN after adding ZIF8 nanoparticles (20 µg/mL). After 7 days, these cells were treated and analyzed as above.

### Immunofluorescence staining

DPSCs were incubated in B-27 Plus Neuronal Culture System, and treated with various concentrations of ZIF-8 or ZnCl_2_. After 7 days, these cells were rinsed with PBS, fixed with 4% paraformaldehyde, permeabilized with 0.2% Triton X-100, and blocked with 2% BSA. Subsequently, the samples were incubated with anti-βIII-tubulin primary antibody (Abcam, ab7260, USA) and anti-NeuN primary antibody (Abcam, ab78078, USA), then incubated with corresponding FITC-labeled goat anti-mouse and Cy3-labeled goat anti-rabbit secondary antibody (Servicebio, China), and finally stained with DAPI (Servicebio, China). The fluorescence distribution of these cells was observed by fluorescence microscopy.

### Transwell migration assay

To investigate the effect of ZIF-8 nanoparticles on DPSCs promoting angiogenesis, 1 × 10^4^ Human Umbilical Vein Endothelial Cells (HUVECs, within 6 generations, Lonza) were resuspended in 100 µL supernatant from DPSC treated with different concentrations of ZIF-8 with or without TPEN for 3 days and seeded in the upper chamber of a 24-well Transwell nest (Corning, 3422). Meanwhile, serum-free medium was added into the lower chamber. After 24 h, the cells on the upper surface were scraped off, then the chamber was fixed with 4% paraformaldehyde for 30 min, stained with 0.1% crystal violet for 15 min, and photographed under a microscope.

### Enzyme-linked immunosorbent assay (ELISA)

2 × 10^6^ DPSCs were seeded in 6-well plates and treated with different concentrations of ZIF-8 (0–50 µg/mL). After 7 days of treatment, the supernatants were collected and subjected to an ELISA assay for VEGF-a as prescribed by the manufacturer (Servicebio, China).

### Transcriptome analysis

1 × 10^7^ DPSCs were incubated in B-27 Plus Neuronal Culture System, treated with 20 µg/mL ZIF-8 for 7 days. Total RNA was extracted by TRIzol Reagent (Invitrogen, cat). RNA quality was determined by testing A260/A280 with Nanodrop™ Onec spectrophotometer (Thermo Fisher Scientific, Inc.). RNA integrity was confirmed by 1.5% agarose gel electrophoresis. Qualified RNAs were finally quantified by Qubit3.0 and QubitTM RNA Wide Range Assay Kit (Life Technologies, Q10210). Strand RNA sequencing libraries were prepared using KCTM stranded mRNA library preparation kit for Illumina (Catalog NO. 2, Wuhan Saikang Medical Co., LTD DR08402 China) in accordance with the manufacturer’s instructions. PCR products corresponding to 200–500 bps were enriched, quantified and sequenced on DNBSEQ-T7 sequencer (MGI technology Co., Ltd.). Model PE150. RNA-Seq data analysis Raw sequencing data were first filtered by Trimmomatic (version 0.36), low-quality reads were discarded, and adaptor sequence contaminated reads were pruned. Reads in the exon region of each gene were counted by featurerts (Subread-1.5.1; Bioconductor) and then calculate the RPKMs. Limma packets were used to identify genes that were differentially expressed between groups. A p-value cutoff of 0.05 and a fold-change cutoff of 1.5 were used to judge the statistical significance of differenced gene expressions (DEGs). Gene ontology (GO) analysis and Kyoto encyclopedia of genes and genomes (KEGG) enrichment analysis for DEGs were both implemented by DAVID (https://david.ncifcrf.gov) with a p-value cutoff of 0.05 to judge statistically significant enrichment.

### Western blot analysis

DPSCs were treated with a series of concentrations of ZIF-8 to detect the induction of neural differentiation. In addition, TPEN was used to verify the roles of zinc ions. SP600125 (Aladdin, S125267), a JNK pathway inhibitor, and SB203580 (MedChemExpress, HY-10,256), a p38 MAPK inhibitor, were used to explore the internal mechanism. After neural induction for 7 days, proteins were extracted with radioimmunoprecipitation assay (RIPA) lysis containing phenylmethylsulfonyl fluoride (PMSF) and phosphorylated protease inhibitors and their concentrations were determined by the BCA Protein Assay Kit (Beyotime, Beijing, China). These protein samples were separated by sodium dodecyl sulfate-polyacrylamide gels (SDS-PAGE) electrophoresis and transferred to polyvinylidene fluoride (PVDF) membranes. After blocking, the membranes were incubated overnight at 4℃ with primary antibody including anti-Nestin (Santa Cruz, sc-23,927, 1:200, USA), anti-βIII-tubulin (Servicebio, GB12139, 1:1000, China), anti-GFAP (Santa Cruz, sc-33,673, 1:200, USA), anti-NeuN (Servicebio, GB11138, 1:1000, China), anti-S100b (Santa Cruz, sc-58,839, 1:200, USA), anti-VEGF-a (Servicebio, GB11034b, 1:1000, China), anti-ERK1/2 (Cell Signaling Technology, 4695P, 1:500, USA), anti-p-ERK1/2 (Cell Signaling Technology, 4370P, 1:500, USA), anti-JNK1 (Cell Signaling Technology, 9252P, 1:500, USA), anti-p-JNK1 (Cell Signaling Technology, 9255 S, 1:500, USA), anti-p38 (Cell Signaling Technology, 8690P, 1:500, USA), anti-p-p38 (Cell Signaling Technology, 4511 S, 1:500, USA), anti-GAPDH (Servicebio, GB15002, 1:1000, China), anti-β-actin (Servicebio, GB15001, 1:1000, China). The next day, after incubation with the corresponding secondary antibody, the bands were visualized using enhanced chemiluminescence substrates.

### Animals and ethics statement

Thirty female Sprague-Dawley rats (4 weeks old) were purchased from the Experimental Animal Center of Three Gorges University (Yichang, Hubei, China) and adaptively fed with adequate diet in a suitable environment for one week. All surgical procedures were approved by the Experimental Animal Ethics Committee of Renmin Hospital of Wuhan University (20220704B).

### Surgical procedure and treatment

The animal studies were reviewed by the Ethics Research Committee of the Renmin Hospital of Wuhan University (20220704B). To establish the SCI model, rats were anesthetized by intraperitoneal injection of 3% phenobarbital (0.1 mL/100 g), and then T10 laminectomy was conducted to expose the spinal cord. Except for the sham operation group, the spinal cord was compressed by a vascular clamp with a closing force of 50G for 15 s, and the tail of rats swung from side to side and then suddenly drooped, which was considered as a sign of successful SCI model establishment. The muscle, fascia and skin were sutured layer by layer. After surgery, penicillin was injected intramuscularly for 3 days, and the bladder was manually emptied twice daily until the urinary function was restored.

All rats were randomly assigned to 5 groups (n = 6): (1) sham operation group, (2) SCI model group, (3) GelMA, (4) Con-DPSCs, and (5) ZIF-8-DPSCs. Rats in the sham operation group only received laminectomy without spinal cord compression. DPSCs were treated with or without ZIF-8 (25 µg/mL) for 24 h, and resuspended in GelMA hydrogel (5 × 10^7^cells/mL) for the ZIF-8-DPSCs group and Con-DPSCs group. At 24 h post injury, 10µL GelMA hydrogel with or without cells was injected into the spinal cord injury site by stereotaxic instrument and microinjection pump, and solidified with a light curing lamp for 10s. The SCI model group was treated with an equal volume of saline as the control.

### Behavioral tests

The spinal cord function in rats was evaluated by video recordings, Basso, Beattie & Bresnahan (BBB) locomotor rating scale, oblique plate test, and footprint analysis on days 7,14,21, and 28 after SCI. The rats were placed in an open field and walked along the wall, and videos were taken to analyze lower limb activity. BBB scores were performed by three trained observers who were blinded to the experimental design. After painting the hind paws with red ink, the rats were asked to run in a narrow dark track (10 cm x 100 cm) lined with paper, which was collected and used for footprint analysis. For the slope test, the rats were placed on an angle-adjustable inclined plate. The angle between the inclined plate and the desktop was slowly increased until the rat stayed in the original position for about 5s, recording the angles.

### H&E staining

On Day 28 after SCI, the rats were perfused with saline and 4% paraformaldehyde. The spinal cord around the injury site (2 cm in length) and vital organs were carefully separated and fixed in 4% paraformaldehyde, and then made into paraffin Sect. (5 µM thickness). The sections were stained with hematoxylin and eosin (H & E) and photographed under an optical microscope.

### Nissl staining

After dewaxing and rehydration, paraffin slices were incubated with Nissl staining solution (Beyotime, C0117) at 37 °C for 3 min, and then washed with distilled water and 95% ethanol according to the instructions. After dehydration, transparency and sealing, images were obtained under a microscope (Nikon), and the number of neurons was counted by the ImageJ software (Media Cybernetics).

### Immunohistochemical staining

Paraffin sections were baked at 60 °C for 30 min, dewaxed with xylene and ethanol, and then rehydrated. Endogenous peroxidase was inactivated by incubation with 3% H_2_O_2_ for 15 min. The sections were soaked with 0.01 M sodium citrate buffer solution (pH = 6.0) at high pressure for 5 min to repair the antigen, followed by cooling naturally. Subsequently, the sections were blocked with 1% bovine serum albumin (BSA) at room temperature for 1 h, and incubated with primary antibody overnight at 4 °C. Afterwards, the samples were incubated with the corresponding secondary antibody at room temperature for 1 h and stained with DAPI. Images were collected using an upright microscope and analyzed using ImageJ (National Institutes of Health, USA).

### Serum biochemical tests

After 28 days of treatment, the rats were sacrificed and their serum and major organs were harvested. Blood biochemical indexes were determined: aspartate alanine aminotransferase (ALT), aminotransferase (AST), creatinine (CRE), serum urea (UREA), and serum zinc.

### Statistical analysis

All experiments in this paper were repeated three times. Data were expressed as Mean and standard deviation (Mean ± SD). Statistical analysis of experimental data was performed using one-way ANOVA and Student Newman Keuls test for multiple comparisons in PRISM8 software (USA) (*P < 0.05, **P < 0.01, and ***P < 0.001).

### Electronic supplementary material

Below is the link to the electronic supplementary material.

Supplementary Material 1

## Data Availability

All data are available in the main text or the supplementary materials.
