# Correction: Metal-organic framework materials promote neural differentiation of dental pulp stem cells in spinal cord injury

**DOI:** 10.1186/s12951-023-02141-5

**Published:** 2023-10-24

**Authors:** Heng Zhou, Shuili Jing, Wei Xiong, Yangzhi Zhu, Xingxiang Duan, Ruohan Li, Youjian Peng, Tushar Kumeria, Yan He, Qingsong Ye

**Affiliations:** 1 Center of Regenerative Medicine, Department of Stomatology, Renmin Hospital of Wuhan University, 430060 Wuhan, China; 2 Terasaki Institute for Biomedical Innovation, 90095 Los Angeles, CA USA; 3 Institute of Regenerative and Translational Medicine, Tianyou Hospital of Wuhan University of Science and Technology, 430064 Wuhan, Hubei China; 4 Department of Oral and Maxillofacial Surgery, Massachusetts General Hospital, Harvard Medical School, 02114 Boston, MA USA; 5 School of Materials Science and Engineering, University of New South Wales, Sydney, NSW Australia

**Correction: Journal of Nanobiotechnology (2023) 21: 316**

10.1186/s12951-023-02001-2

In this article Heng Zhou, Shuili Jing and Wei Xiong should have been denoted as an equally contributing authors.

In addition, the affiliation details for Yan He were incorrectly given as: 3 Institute of Regenerative and Translational Medicine, Tianyou Hospital of Wuhan University of Science and Technology, Wuhan 430064, Hubei, China 4 Department of Oral and Maxillofacial Surgery, Massachusetts General Hospital, Harvard Medical School, Boston, MA 02114, USA.

But should have been 1 Center of Regenerative Medicine, Department of Stomatology, Renmin Hospital of Wuhan University, Wuhan 430060, China, 3 Institute of Regenerative and Translational Medicine, Tianyou Hospital of Wuhan University of Science and Technology, Wuhan 430064, Hubei, China, 4 Department of Oral and Maxillofacial Surgery, Massachusetts General Hospital, Harvard Medical School, Boston, MA 02114, USA.

 The authors apologise for this error. The original article [1] has been revised.
