# Supplementary material for: Metal-organic framework materials promote neural differentiation of dental pulp stem cells in spinal cord injury
Source: J Nanobiotechnology. 2023 Sep 4;21:316. doi: 10.1186/s12951-023-02001-2 (PMC10478386; doi:10.1186/s12951-023-02001-2)
Supplement: Supplementary file 1 — Supplementary Material 1 [file 12951_2023_2001_MOESM1_ESM.docx]

**Metal-organic framework materials promote neural differentiation of dental pulp stem cells in spinal cord injury**

Heng Zhou^1,#^, Shuili Jing^1^^,#^, Wei Xiong^1,#^, Yangzhi Zhu^2^, Xingxiang Duan^1^, Ruohan Li^1^, Youjian Peng^1^, Yan He^3,4*^, Qingsong Ye^1,4*^

1 Center of Regenerative Medicine & Department of Stomatology, Renmin Hospital of Wuhan University, Wuhan, 430060, China

2 Terasaki Institute for Biomedical Innovation, Los Angeles, CA, 90095, USA

3 Institute of Regenerative and Translational Medicine, Tianyou Hospital of Wuhan University of Science and Technology, Wuhan, Hubei, 430064, China

4 Department of Oral and Maxillofacial Surgery, Massachusetts General Hospital, Harvard Medical School, Boston, 02114, MA, USA

*Corresponding authors:

*Qingsong Ye

Center of Regenerative Medicine, Renmin Hospital of Wuhan University, Wuhan, China. Email: [qingsongye@whu.edu.cn](mailto:qingsongye@whu.edu.cn)

*Yan He

Institute of Regenerative and Translational Medicine, Tianyou Hospital of Wuhan University of Science and Technology, Wuhan, China. Email: [helen-1101@hotmail.com](mailto:helen-1101@hotmail.com)

**Supplemental Materials**

**
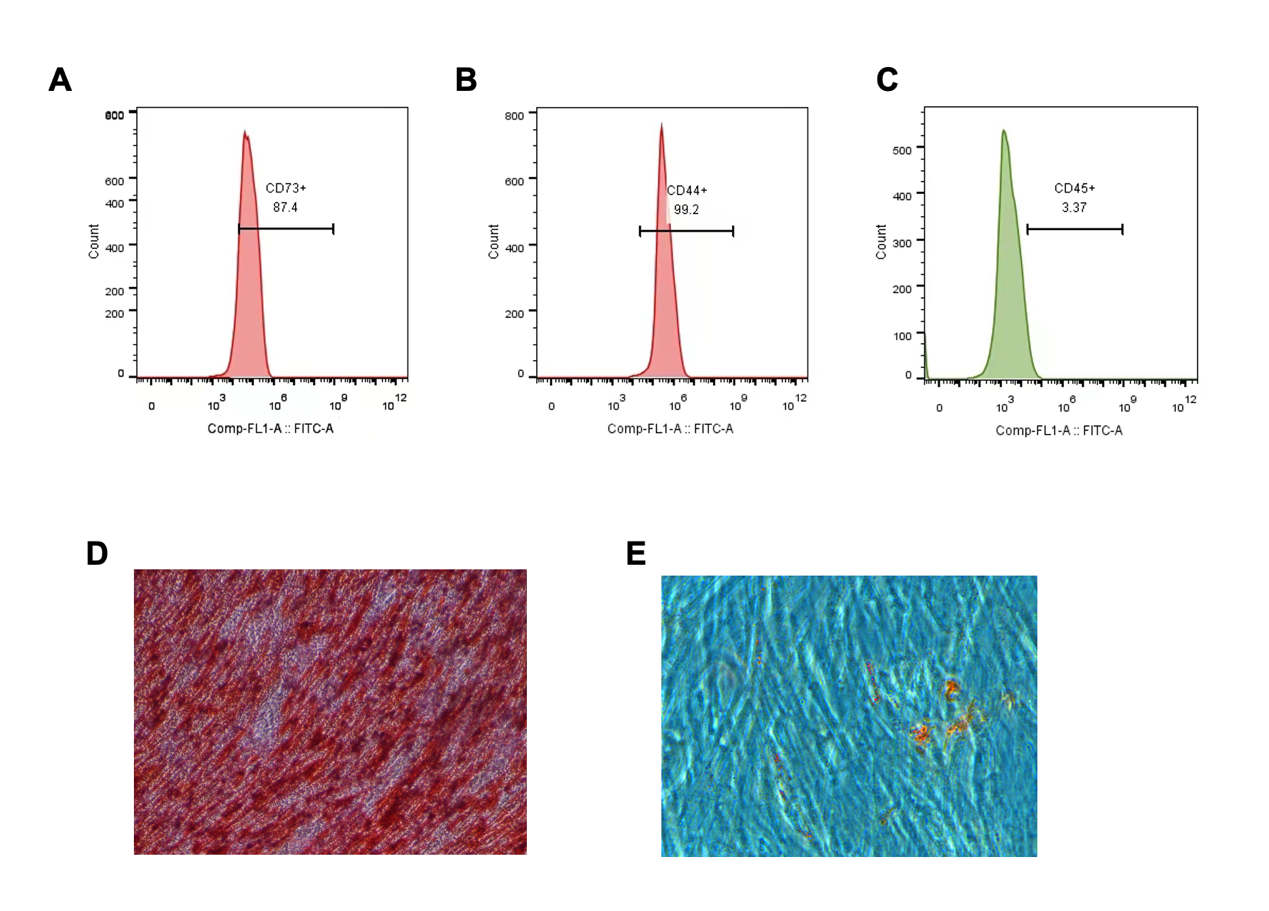
**

**Supplemental Figure 1. Identification of dental pulp stem cells.** Dental pulp stem cells were extracted and cultured into P3. Flow cytometry was used to detect (A) CD73, (B) CD44 and (C) CD45. (D) The matrix mineralization of DPSCs was detected by alizarin red staining after 14 days of osteogenic differentiation culture. (E) Lipid differentiation culture for 14 days, oil red O staining was used to detect fat droplets.


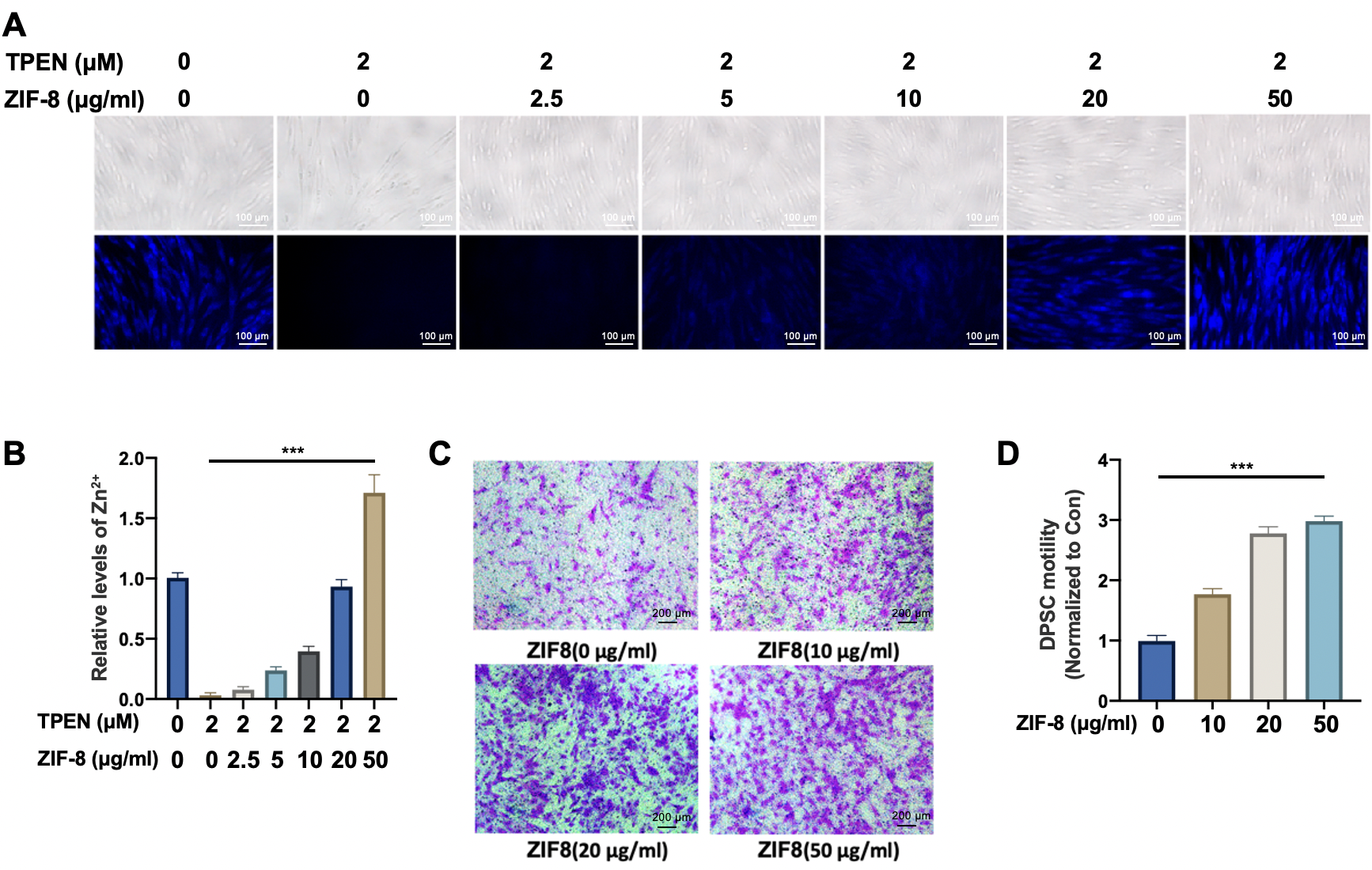


**Supplemental Figure 2. ZIF-8 reverses Zn deprivation induced by TPEN and promotes DPSCs cell migration.** (A) DPSCs were treated with TPEN (0 and 2 μM) combined with ZIF-8 of concentration gradient (0, 2.5, 5, 10, 20 and 50 μg/ml). TSQ fluorescence staining was used to detect Zn^2+^ in DPSCs. (B) Fluorescence intensity was quantified. (C) DPSCs were treated with with ZIF-8 (0, 10, 20 and 50 μg/ml). Then cells stained with the crystal violet stain for Transwell experiments. (D) The number of transferred cells is quantified. *** P < 0.001.


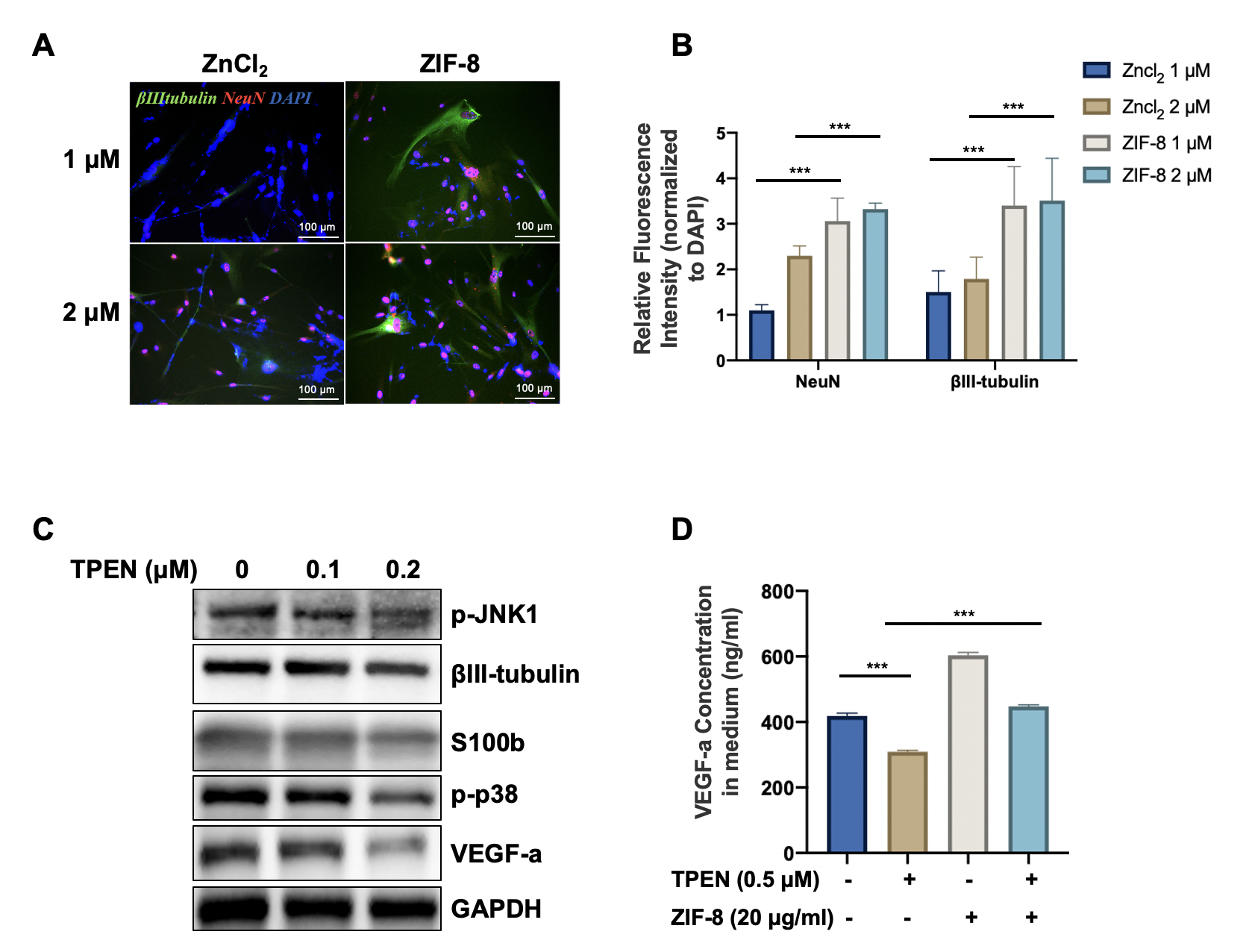


**Supplemental Figure 3. Zn^2+^ is associated with neural differentiation and angiogenesis of DPSCs.** (A) DPSCs were treated with TPEN (0, 0.1 and 0.2 μM). Western Blot was used to detect the expression of p-JNK1, p-38, VEGF-a, βIII-tubulin, and GAPDH. (B) DPSCs were treated with same molar concentrations of Zncl_2_ or ZIF-8 (1 and 2 μM) in B27 neural differentiation medium for 7 days. Immunofluorescence analysis of expression of βIII-tubulin (green) and NeuN (red). (C) DPSCs were treated with TPEN (0 and 0.5 μM) combined with ZIF-8 (0 and 20 μg/ml). VEGF-a in the medium was quantified by ELISA. *** P < 0.001.


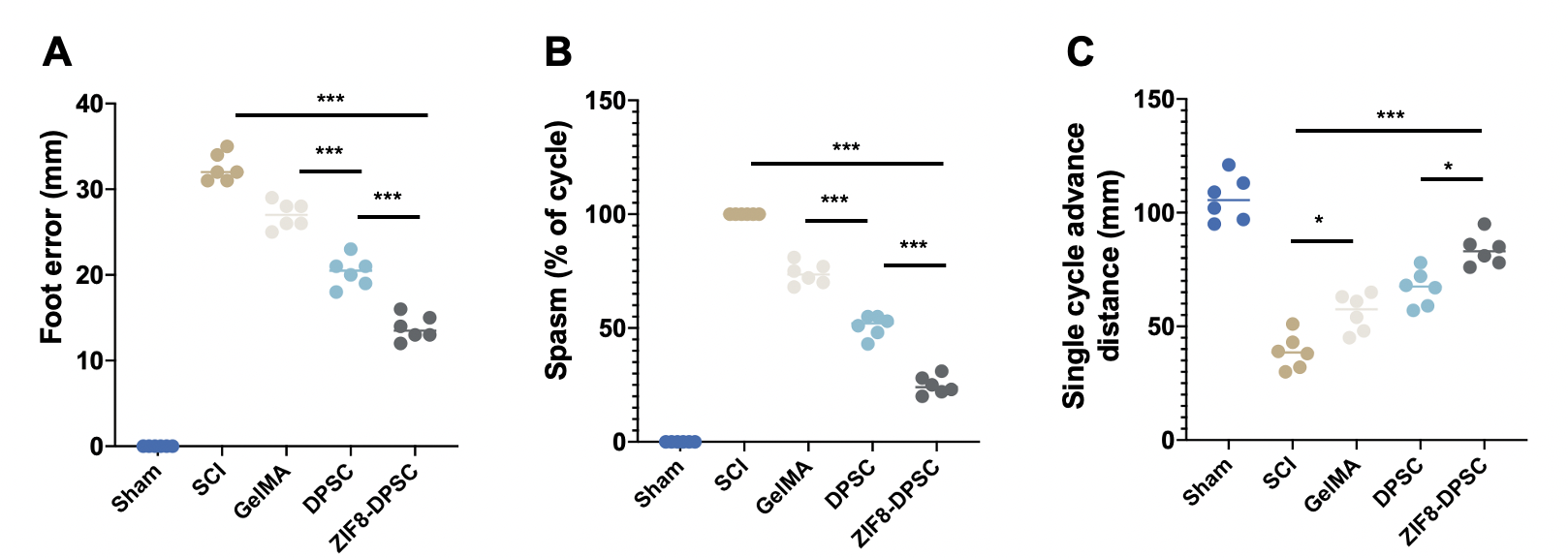


**Supplemental Figure 4. ZIF-8-DPSCs promote the recovery of motorial function in SCI rats.** (A) Foot error. (B) Spasm. (C) Single cycle advance distance of the rats in the Sham, SCI group, GelMA group, DPSCs group and ZIF-8-DPSCs group were analyzed. **P < 0.01. *** P < 0.001. n.s. means no statistical significance. Data are represented as mean ± SD (n = 6).


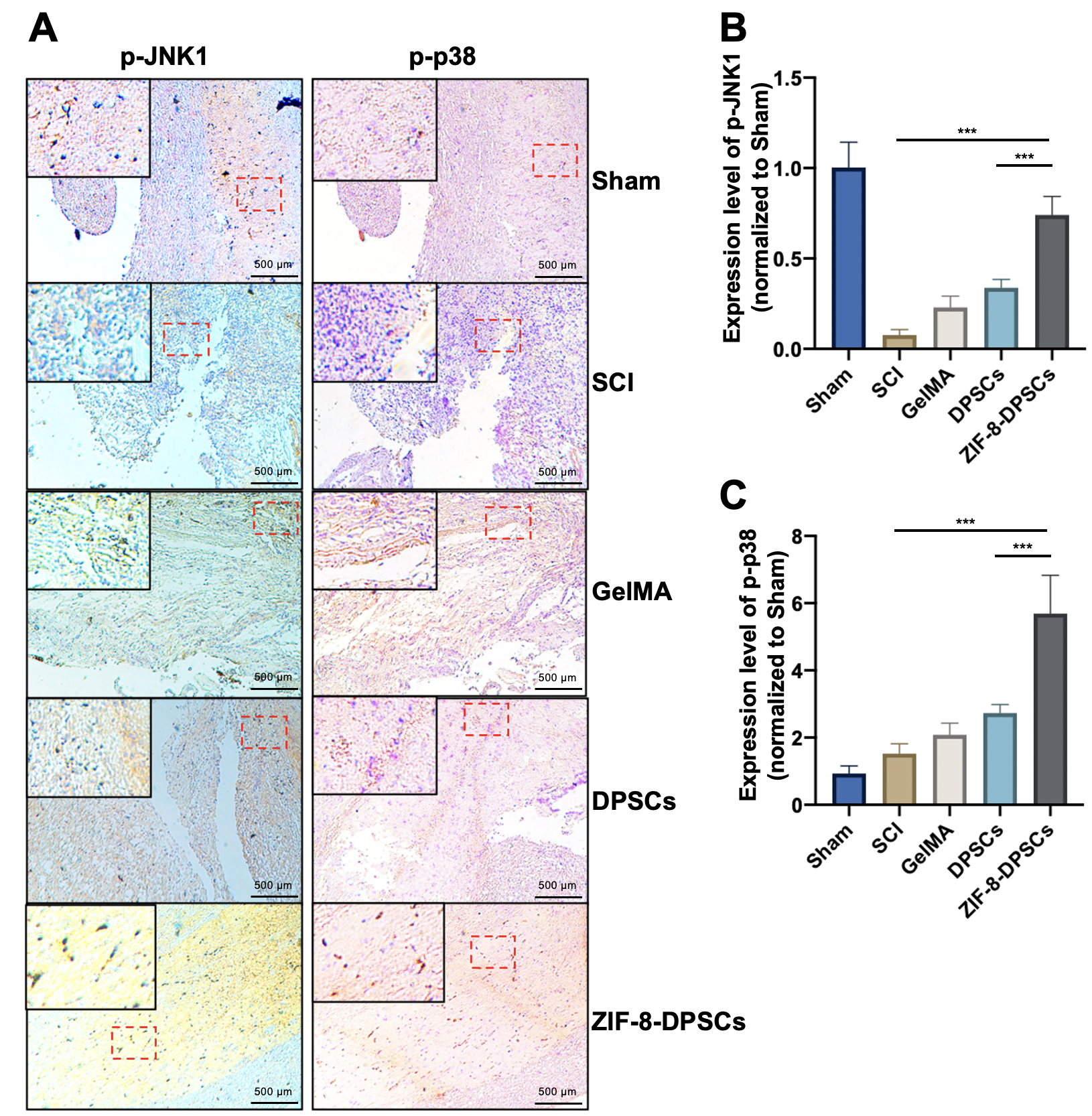


**Supplemental Figure 5. ZIF-8-DPSCs promote activating of MAPK signaling pathway in SCI rats.** (A) IHC was used to detect the expression of p-JNK1 and p-p38 in the Sham, SCI group, GelMA group, DPSCs group and ZIF-8-DPSCs group. The expression of (B) p-JNK1 and (C) p-p38 were quantified. **P < 0.01. *** P < 0.001. n.s. means no statistical significance. Data are represented as mean ± SD (n = 6).


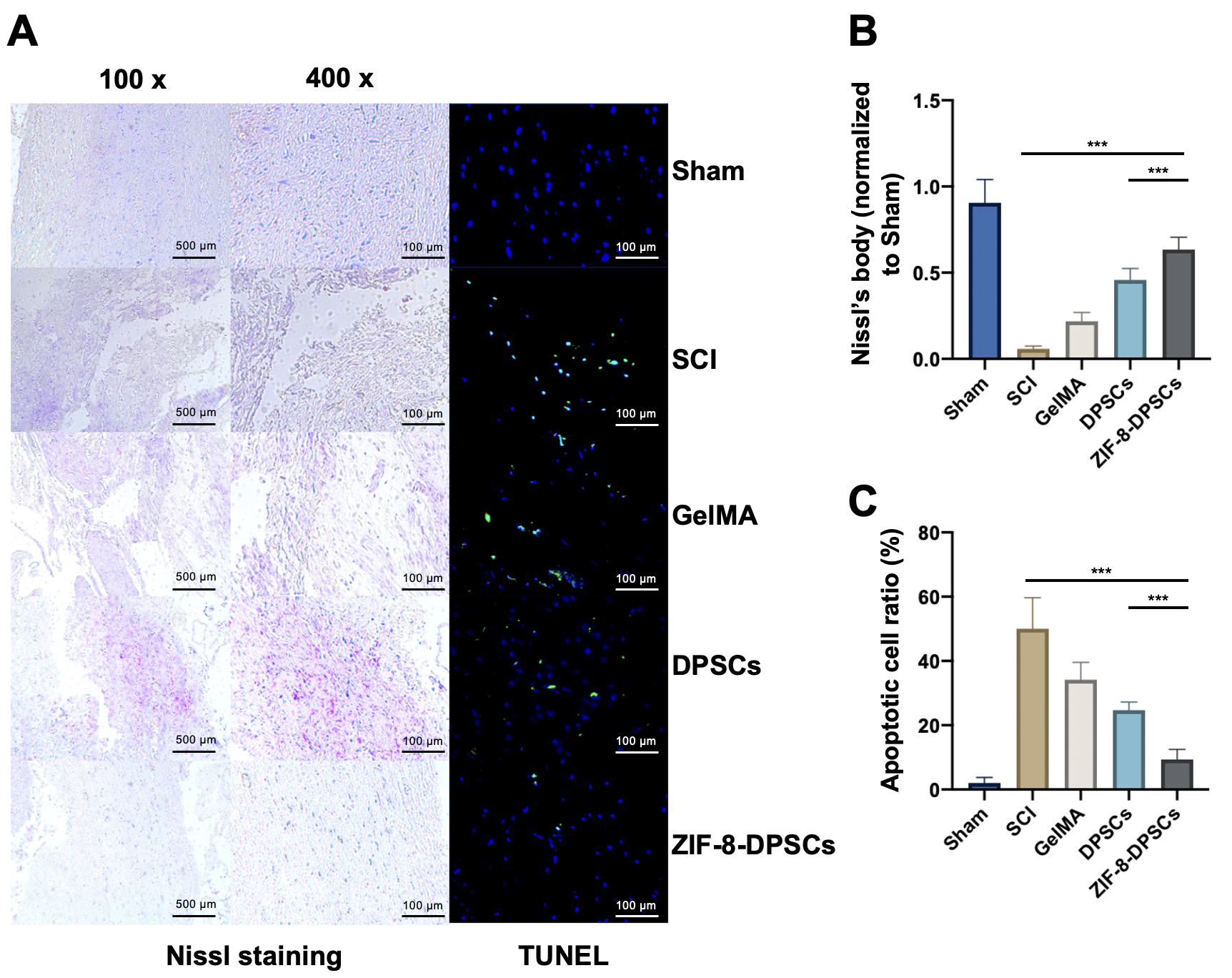


**Supplemental Figure 6. ZIF-8-DPSCs promoted nerve regeneration and inhibited apoptosis n SCI rats.** (A) Nissl staining and TUNEL staining were used to detect nerve regeneration and apoptosis. in the Sham, SCI group, GelMA group, DPSCs group and ZIF-8-DPSCs group. The area of Nissl bodies and the proportion of apoptotic cells were quantified. *** P < 0.001.


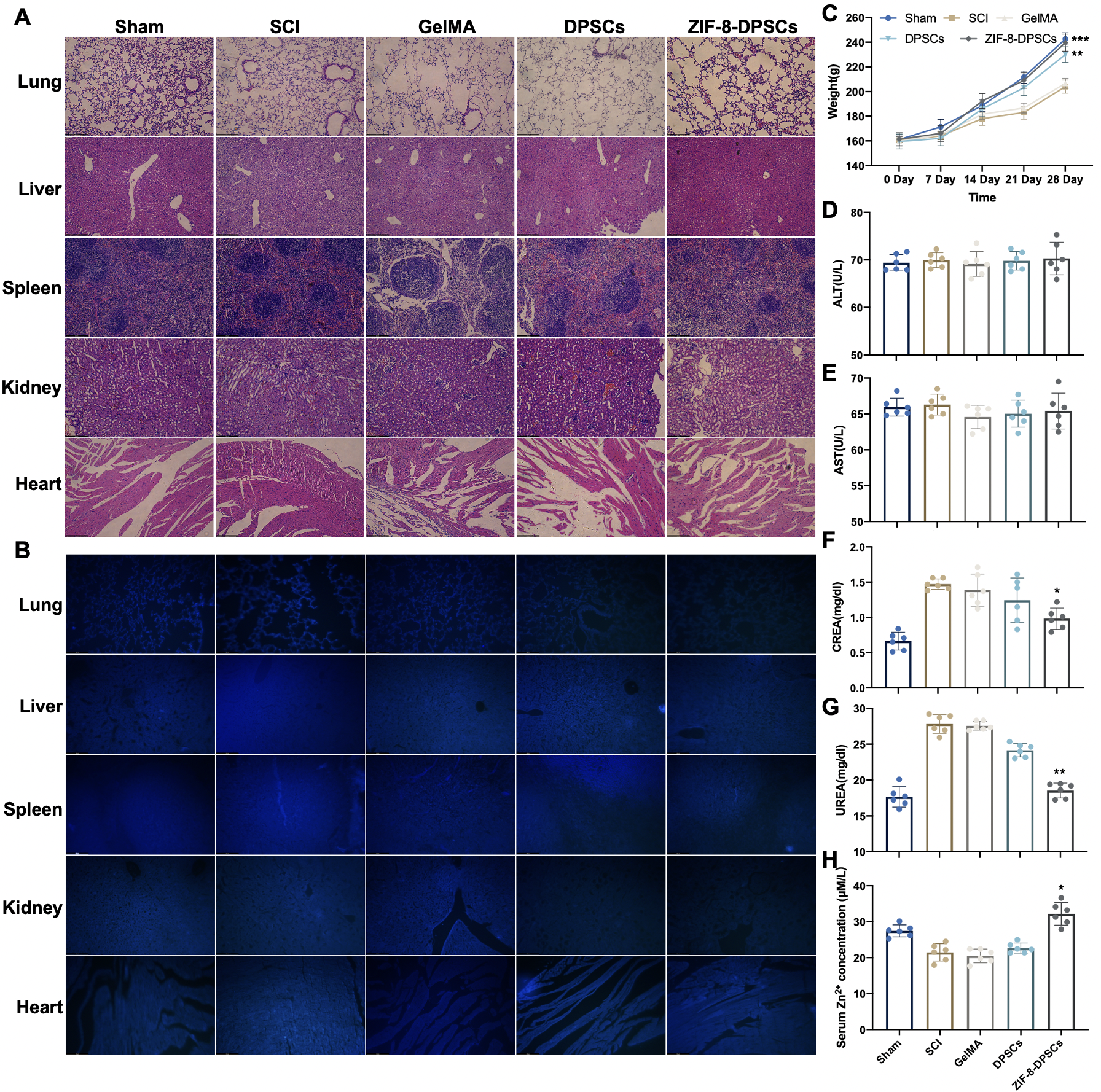


**Supplemental Figure 7. Biocompatibility test in Vivo.** (A) H&E and (B) TSQ staining of major organs (lung, liver, spleen, kidney and heart) in rats treated with different strategies. (C) Body weights of rats in each group. Blood biochemistry analysis of (D) ALT, (E) AST, (F) CREA, (G) UREA, and (H) Serum Zn^2+^ levels. *P <0.05. **P < 0.01. *** P < 0.001. vs. SCI group. Data are represented as mean ± SD (n = 6).
